# Biological methane production and accumulation under sulfate-rich conditions at Cape Lookout Bight, NC

**DOI:** 10.3389/fmicb.2023.1268361

**Published:** 2023-10-06

**Authors:** Gage R. Coon, Paul D. Duesing, Raegan Paul, Jennifer A. Baily, Karen G. Lloyd

**Affiliations:** Department of Microbiology, The University of Tennessee, Knoxville, TN, United States

**Keywords:** marine sediments, methanogenesis, sulfate reduction, AOM, hydrogen, thermodynamics

## Abstract

**Introduction:**

Anaerobic oxidation of methane (AOM) is hypothesized to occur through reverse hydrogenotrophic methanogenesis in marine sediments because sulfate reducers pull hydrogen concentrations so low that reverse hydrogenotrophic methanogenesis is exergonic. If true, hydrogenotrophic methanogenesis can theoretically co-occur with sulfate reduction if the organic matter is so labile that fermenters produce more hydrogen than sulfate reducers can consume, causing hydrogen concentrations to rise. Finding accumulation of biologically-produced methane in sulfate-containing organic-rich sediments would therefore support the theory that AOM occurs through reverse hydrogenotrophic methanogenesis since it would signal the absence of net AOM in the presence of sulfate.

**Methods:**

16S rRNA gene libraries were compared to geochemistry and incubations in high depth-resolution sediment cores collected from organic-rich Cape Lookout Bight, North Carolina.

**Results:**

We found that methane began to accumulate while sulfate is still abundant (6–8 mM). Methane-cycling archaea *ANME-1*, *Methanosarciniales*, and *Methanomicrobiales* also increased at these depths. Incubations showed that methane production in the upper 16 cm in sulfate-rich sediments was biotic since it could be inhibited by 2-bromoethanosulfonoic acid (BES).

**Discussion:**

We conclude that methanogens mediate biological methane production in these organic-rich sediments at sulfate concentrations that inhibit methanogenesis in sediments with less labile organic matter, and that methane accumulation and growth of methanogens can occur under these conditions as well. Our data supports the theory that H_2_ concentrations, rather than the co-occurrence of sulfate and methane, control whether methanogenesis or AOM via reverse hydrogenotrophic methanogenesis occurs. We hypothesize that the high amount of labile organic matter at this site prevents AOM, allowing methane accumulation when sulfate is low but still present in mM concentrations.

## 1. Introduction

Atmospheric methane concentrations from all sources have more than doubled from 0.7 to 1.9 ppmv since the pre-industrial era (Etheridge et al., 1998; US Department of Commerce, 2023). Methane contributes 20% of total greenhouse gas emissions, largely originating from the microbial process of methanogenesis (Wuebbles and Hayhoe, 2002). Methanogenesis has three main pathways: hydrogenotrophic using hydrogen and carbon dioxide, acetoclastic using acetate, and methylotrophic using methylated compounds, with hydrogenotrophic methanogenesis often being the most prevalent type in marine sediments (Liu and Whitman, 2008). All types of methanogenic pathways use the methyl coenzyme M reductase (MCR) enzyme to catalyze the final step of methane production. The same gene is used in reverse methanogenesis, also called the anaerobic oxidation of methane (AOM), as well (Hallam et al., 2004; Scheller et al., 2010; Soo et al., 2016; Yu et al., 2022). In many types of marine sediments, more than eight times more methane is biotically produced in the sediment than is released into the overlying water (Reeburgh et al., 1991; Mau et al., 2017; Ruffine et al., 2018). This disparity is predominantly due to sulfate-dependent AOM, which oxidizes much of the methane to carbon dioxide before it can escape the sediment column (Reeburgh, 2007), though AOM can also rely on other electron acceptors like nitrate, nitrite, and metal ions (Muyzer and Stams, 2008; Beal et al., 2009; Haroon et al., 2013; Timmers et al., 2017; Zhang et al., 2022).

In marine sediment, sulfate-dependent AOM can occur through either reverse hydrogenotrophic methanogenesis coupled to sulfate reduction, also known as interspecies hydrogen transfer (Hoehler et al., 1994, 1998; Timmers et al., 2017) or via direct interspecies electron transfer to a sulfate reducer (McGlynn et al., 2015; Wegener et al., 2015). AOM defines methane dynamics in marine sediment, so understanding the role of these two different mechanisms is key. Evidence for reverse hydrogenotrophic methanogenesis coupled to sulfate reduction comes from the fact that net methane oxidation only occurs when hydrogen concentrations drop low enough to make reverse hydrogenotrophic methanogenesis sufficiently exergonic to meet microbial energy demands (Hoehler et al., 1994). Hydrogen has been shown to control the direction of methane production or oxidation in enrichments of methanogen-like archaea *Methanosarciniales* (*ANME-2*) and *ANME-1* (Yoshinaga et al., 2014; Wegener et al., 2015). Pure cultures of methanogens produce hydrogen from methane while under low-hydrogen conditions, though they do not sustain this process at a high rate for more than a few hours (Valentine et al., 2000). *Methanosarcina barkeri*, another methanogen, has recently been characterized as capable of AOM (Yu et al., 2022). On the other hand, evidence that the mechanism of AOM occurs through direct electron transfer, independently of a molecular intermediate comes from the presence of nanowire-like appendages on *ANME-1* (Wegener et al., 2015) and multiheme cytochromes on consortia of the *Methanosarciniales* (*ANME-2*) that have been shown to conduct electricity (McGlynn et al., 2015). *ANME-2* exhibits the use of artificial electron acceptors for methanotrophy (Scheller et al., 2016), suggesting that sulfate is not necessary as the sink for electrons from methane oxidation, though some mean of transporting electrons is necessary. These studies with direct electron transfer were conducted in deeply-sourced methane seeps, so they deserve more study in coastal marine sediments.

If the mechanism of the apparent sulfate-driven control of methane is due to the balance between biological hydrogen production and consumption, rather than direct electron transfer from methane to sulfate, then AOM and methanogenesis should be decoupled from the presence of sulfate in areas of high organic matter content where hydrogen supply can overwhelm sulfate reduction. In one example of this, high concentrations of fermentative products in highly labile sludge reactors support simultaneous sulfate reduction and methanogenesis (Santegoeds et al., 1999). Additionally, incubations of marine sediments show methane production occurring with sulfate present, even when methane does not accumulate (Timmers et al., 2015; Sela-Adler et al., 2017; Xiao et al., 2017; Maltby et al., 2018; Kevorkian et al., 2022). AOM and methanogenesis should also occur nearly simultaneously when hydrogen concentrations get low, regardless of the organic matter content, since pockets of sulfate depletion may allow hydrogen to increase in microenvironments, supporting ephemeral methanogenesis even during AOM (Knab et al., 2008). In support of this, radioactive tracer experiments have shown hydrogenotrophic methane production at depths where AOM prevents methane accumulation (Hoehler et al., 1994; Parkes et al., 2007; Beulig et al., 2019; Krause et al., 2023). Biotic methane production has been shown to occur in the upper few cm of marine sediments in the presence of abundant sulfate (Xiao et al., 2017, 2018). This is partly due to methylotrophic methanogens which do not have to compete with sulfate reducers (Xiao et al., 2018), yet hydrogenotrophic methanogens also are present, suggesting that hydrogenotrophic methanogenesis also contributes (Xiao et al., 2017). The MCR-containing microorganisms present in sulfate-rich non-methane-accumulating sediments have been found to be diverse, spanning many genera within *Methanomicrobiales*, *Methanosarciniales*, and *ANME-1* (Lloyd et al., 2011; Kevorkian et al., 2021; Krause et al., 2023). Cultured representatives of the *Methanomicrobiales* generally use hydrogen with carbon dioxide or formate, and those from the Methanosarciniales can also use acetate and methylated compounds, some obligately so. There are no pure cultures from *ANME-1*, but enrichments and experiments on natural sediments suggest they may alternate between AOM and hydrogenotrophic methanogenesis (Lloyd et al., 2011; Kevorkian et al., 2021; Krause et al., 2023).

To help distinguish between the two mechanisms, we examined downcore geochemistry and microbiology in marine sediments. Methane usually does not accumulate in marine sediments until a depth where sulfate, which diffuses into sediments from the overlying water, is depleted (Reeburgh, 2007). This is due to two reasons: (1) sulfate-reducing bacteria keep the hydrogen and acetate produced by fermentation of organic matter low enough that most methanogenesis is thermodynamically inhibited, and (2) AOM occurs either because sulfate reducers pull hydrogen concentrations low enough that reverse hydrogenotrophic methanogenesis is exergonic or because anaerobic methanotrophs pass electrons directly from methane to sulfate (Reeburgh, 2007; Larowe et al., 2008). Below the depth where sulfate is consumed, hydrogen increases, methanogenesis is no longer inhibited, and methane accumulates (Hoehler et al., 2001). Observing methane accumulation due to diffusion from below and no AOM in the presence of sulfate in sediments would provide another piece of support for the theory that AOM occurs through reversible hydrogenotrophic methanogenesis via interspecies hydrogen transfer to a sulfate reducer. Such a result would suggest that methanogenesis and AOM are indirectly dependent on the presence of sulfate, and that AOM can be inefficient, even when sulfate is present. Organic-rich sediments such as those of Cape Lookout Bight (CLB), North Carolina (Hoehler et al., 1994; Martens et al., 1998) and Beidagang Wetland Nature Reserve, China (La et al., 2022) have been shown to lack AOM through radiotracers, sulfate and methane profiles, and stable carbon isotopes, even in the presence of millimolar concentrations of sulfate. In CLB, this is because the hydrogen concentrations do not drop low enough to make reverse methanogenesis exergonic past the minimum energetically profitable ΔG of less than −10 kJ/mol (Hoehler et al., 2001). This happens because the site has a high sedimentation rate (up to 10 cm/yr) of highly labile organic carbon (Martens et al., 1998).

We examined biological methane accumulation in CLB through downcore geochemistry and 16S rRNA gene amplicon surveys, as well as incubations of whole sediments with and without 2-bromoethanesulfonoic acid (BES). BES has been shown to drive *in-vitro* inhibition of the MCR protein (Alperin and Reeburgh, 1985; Webster et al., 2016), therefore inhibiting methane production via BES can show if methane is being actively produced by microorganisms rather than just diffusing out of sediments after diffusing in from elsewhere. Here, we explore the biogeochemistry of these sediments by observing the changes between methane production vs. methane accumulation in sulfate-rich sediments and its relationship to the distribution of bacteria and archaea in organic-rich CLB sediments.

## 2. Methods

### 2.1. Site characteristics

Cape Lookout Bight is a 10 m deep lagoon with brackish water located on the coast of North Carolina. This site has both high organic matter and sediment deposition rates (Chanton et al., 1983; Martens et al., 1992). Sediments have been shown to be anoxic below 2 mm (Canuel and Martens, 1993).

### 2.2. Sample collection

Sediment samples were collected from Cape Lookout Bight, NC (34.6205 N, 76.5500 W) using SCUBA divers June 2021. We collected two large duplicate sediment cores (42 cm) and three small sediment cores (<20 cm) by pushing a polypropylene tube into the sediment and capping the ends of the tube with rubber stoppers. The large cores were sectioned at a 2 cm vertical resolution, where sediment for DNA extraction was placed into cut-off 10 mL syringes, flash frozen in dry ice, and stored at −20°C. 1 mL of sediment was placed in screw cap tubes to measure porosity. For methane measurements, 4 mL of sediment was sampled with cut-off 5 mL plastic syringes and placed in 60 mL glass serum vials containing 1 mL 0.1 M KOH and capped with butyl rubber stoppers. Methane vials were shaken to mix the KOH into the sediment and then stored upside down until measurement to prevent methane from escaping. Sediment was also centrifuged then filtered (0.2 μm) and stored in 1% ZnCl_2_ to measure sulfide and in 1 N HCl to measure sulfate. The three small cores were stored in the tubes used to collect them until used for incubation experiments.

### 2.3. DNA extraction and sequencing

DNA was extracted from 2 g of wet sediment using QIAGEN’s RNeasy Powersoil Total RNA Kit with the RNeasy Powersoil DNA Elution Kit. All steps in the supplied protocols were followed. The V4 region of the 16S rRNA gene was amplified using polymerase chain reaction (PCR) from the Earth Microbiome Project (EMB) 16S Illumina amplicon protocol and Caporaso 515F (GTGCCAGCMGCCGCGGTAA) and 806R (GGACTACHVGGGTWTCTAAT) primers. The amplified 16S rRNA products were prepared using the Illumina DNA prep kit and sequenced using an Illumina MiSeq system.

### 2.4. Data analysis

The sequences were analyzed in R (RStudio Team, 2020; R Core Team, 2021) with the Divisive Amplicon Denoising Algorithm (DADA2) pipeline (Callahan et al., 2016), version 1.22.0, to remove chimeras, control sequence read quality, and trim primers. Samples with poor read quality were removed from the dataset. Contaminants were removed based on the potential contaminant genera identified previously (Sheik et al., 2018) and shown in Supplementary Table S1. Taxonomy was applied by a Naïve Bayes classifier using SILVA reference sequences (Quast et al., 2013; Yilmaz et al., 2014), version 138.1, to identify taxonomy up to the genus level (assignTaxonomy function from the DADA2 package).

The resulting table contained 24,987 ASVs and a total of 4,746,347 reads over 36 samples for an average of 131,843 reads per sample (Supplementary Table S1). The relative abundance of ASVs was calculated based on ASV counts and the sum of total ASV counts. Alpha diversity (Chao 1, Shannon, and Simpson indices) and beta diversity using non-metric multidimensional scaling (NMDS) ordination and Bray–Curtis dissimilarity distances were calculated using the phyloseq package (McMurdie and Holmes, 2013), version 1.38.0.

Plots of taxonomic abundance were plotted using the ggplot2 package (Wickham, 2016) to aid in data visualization. All helper packages and versions are listed in Supplementary Table S2. All raw sequences have been deposited in the NCBI bank under the Accession ID PRJNA949635. All code is available on GitHub at https://github.com/gagecoon/clb21.

### 2.5. Porewater measurements

#### 2.5.1. Porosity

Sediment from the large cores were weighed in plastic screw cap 2 mL tubes. 1 mL of sample was taken from each 2 cm interval sectioned from the core. The screwcap tubes were placed without a lid in an oven for 1 month at 55°C. After 1 month, the samples were weighed, and porosity (Φ) was calculated using the following formula:


$$\Phi =\frac{\left(\frac{m_{w}-m_{d}}{\rho_{pw}}\right)}{\left(\frac{m_{d}}{\rho_{sm}}\right)+\left(\frac{m_{w}-m_{d}}{\rho_{pw}}\right)}$$

Where *m_w_* denotes the wet mass, m_d_ denotes the dry mass, *ρ*_pw_ denotes the porewater density, and ρ_sm_ denotes the solid matter density. Porewater density was assumed to be 1.025 g/cm^3^ and solid matter density was previously measured as 2.34 g/cm^3^ (Alperin, 1988). This procedure was repeated once again after the first results were collected for validation.

#### 2.5.2. Sulfide

Hydrogen sulfide samples were preserved in 1% ZnCl_2_ and measured with an adapted Cline assay for S^−^ measurements. Dilutions of sulfide samples were added to plastic cuvettes with diamine and Fe^3+^ to yield methylene blue (Cline, 1969). The methylene blue product was measured at 667 nm using a NanoDrop 2000 spectrophotometer and corrected for the dilutions used to maintain absorbance between 0.1 and 0.9.

#### 2.5.3. Sulfate

Sulfate tubes were measured via ion chromatography (IC) using a Dionex ICS-2100 system equipped with a 4 mm × 250 mm IonPac AS18 hydroxide-selective anion-exchange column using KOH as the eluent. Single samples ran for 24 min each to allow chloride and sulfate anion separation.

### 2.6. Methane

Methane samples were collected in closed serum vials and measured with a flame ionized detector on a gas chromatograph. Vials were shaken vigorously for 1 min prior to measurement. 0.1 mL of standards and samples were injected into the gas chromatograph in triplicate. Methane concentrations in mM were calculated from the formula:


$$CH_{4_{aq}}=\frac{\mathrm{ppm}*V_{h}}{R*T*\Phi *V_{s}*1000}$$

Where ppm is calculated from the standard curve with the same volume injected into the GC as the samples, *V_h_* is the volume of the headspace (55 mL), R is the universal gas constant (0.082057 L*atm/mol*K), T is the temperature of the site at the time of measurement (295 K), Φ is the sediment porosity, *V_s_* is the volume of the sediment in the sampled serum vial (4 mL), and 1,000 is a conversion factor so concentrations are in mM.

### 2.7. Sediment geochemical analysis

Carbon and nitrogen concentrations (total, organic, and inorganic) and isotopic signatures (δ^13^C and δ^15^N) were measured by grinding dry sediment in a mortar and pestle to create a fine powder. To determine organic content, sediment was subsampled into unacidified sediment and acidified sediment. Acidified sediment was treated with 1 mL of 1 N HCl per 0.3 g sediment. 20–25 mg of dry sediment in tin capsules was measured using a Costech ECS4010 Elemental Analyzer paired to a Thermo-Finnigan Delta + XL mass spectrometer via a Thermo-Finnigan Conflo III. The setup used helium gas and a high temperature, >1,000°C, to analyze the sediment. Organic concentrations of carbon and nitrogen were calculated by the difference between inorganic and total carbon and nitrogen concentrations, respectively.

### 2.8. BES incubation—methanogenesis inhibition

#### 2.8.1. Experiment 1

For Experiment 1, one 23 cm deep core was homogenized and 30 mL of sediment was placed into each of 17 autoclaved 60 mL glass serum vials and capped with thick rubber butyl stoppers. Samples had 5 mL of either 0, 20, or 30 mM of autoclaved 2-bromoethanesulfonic acid (BES) in autoclaved anoxic saline solution (0.29 M NaCl). There were three replicates with 0 mM BES (controls), seven with 20 mM BES, and seven with 30 mM BES, where these concentrations are final volumes accounting for mixing with porewater. Headspaces were gassed out, i.e., sparged with one gas line and two needles, with O_2_-scrubbed N_2_ to create anoxic conditions. These vials were incubated at 37°C while being shaken in the dark. Vials were removed from incubation and the headspace measured for methane and CO_2_ concentrations and methane δ^13^C values periodically. Serum vials were shaken for 1 min before some of the headspace (1–5 mL to keep methane concentrations in range) was injected into a Picarro SSIM2 module, diluted with zero air to be a sufficient volume for the analyzer (100 mL total), and measured for methane and CO_2_ concentrations and methane δ^13^C values on the Picarro G2201-i cavity ring-down spectrometer.

#### 2.8.2. Experiment 2

Experiment 2 was a long-term incubation of two halves of two 16 cm cores – 0–8 cm and 8–16 cm depth sections. Each half of each core was homogenized and 30 mL of sediment was separated into five subsamples, three of which were treated with 5 mL of 20 mM (final volume) autoclaved BES in autoclaved anoxic saline solution (0.29 M NaCl) and two of which were treated with 5 mL of anoxic saline solution to act as controls. This sediment was placed in autoclaved 60 mL glass serum vials and capped with thick rubber butyl stoppers. In total, there were six vials treated with BES for 0–8 cm, three from one core and three from the other, six vials treated with BES for 8–16 cm, three from each core, four treated with no BES for 0–8 cm, two from each core, and four treated with no BES for 8–16 cm, two from each core. These were gassed out, i.e., sparged, with O_2_-scrubbed N_2_ to create anoxic solutions, incubated at 37°C while being shaken in the dark, and measured identically as experiment 1. Serum vials were shaken for 1 min before some of the headspace (1–5 mL to keep methane concentrations in range) was injected into a Picarro SSIM2 module, diluted with zero air to be a sufficient volume for the analyzer (100 mL total), and measured for methane and CO_2_ concentrations and methane δ^13^C values on the Picarro G2201-i cavity ring-down spectrometer.

## 3. Results

### 3.1. Sediment geochemistry and porewater analysis

**Figure 1:**
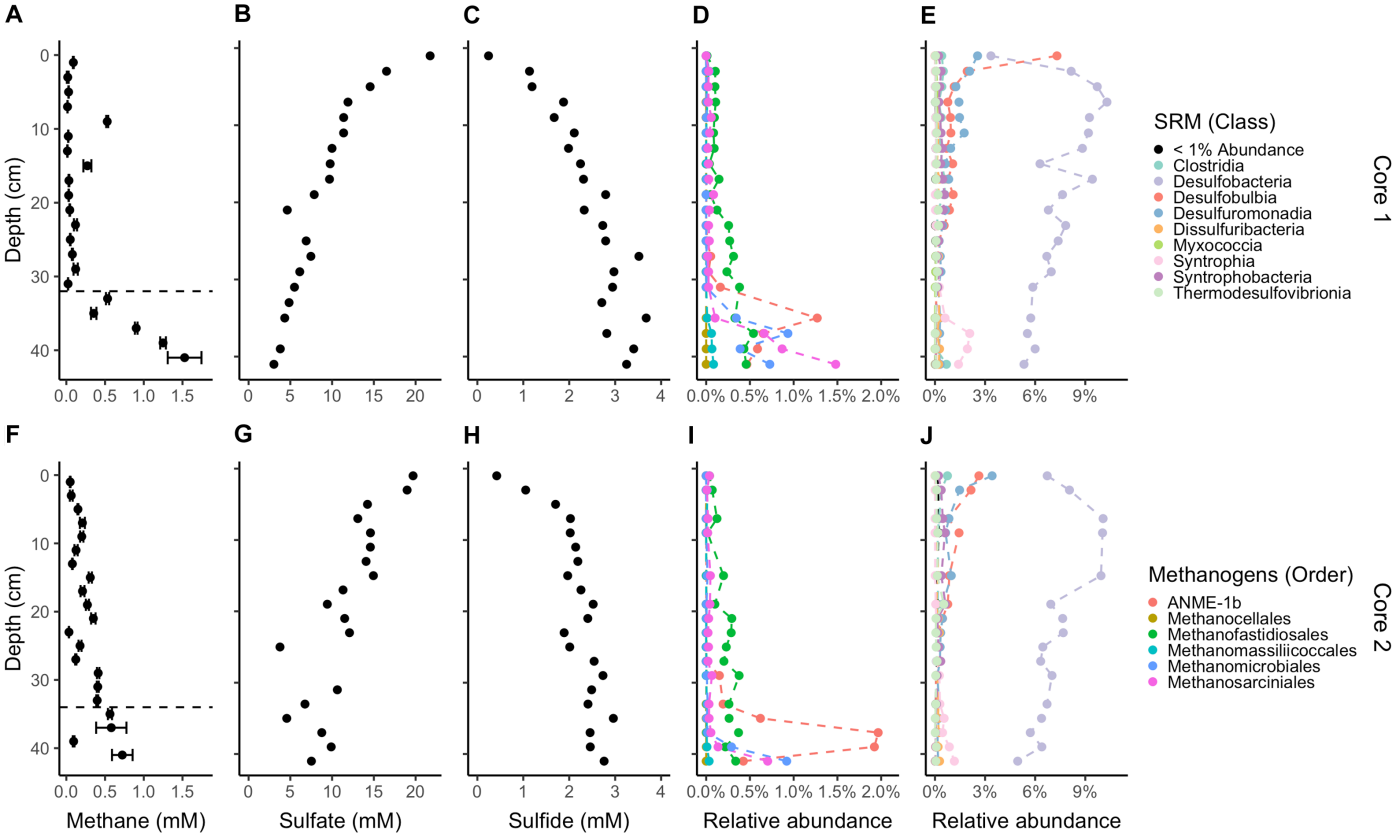
Downcore geochemistry and microbial composition. Concentrations of methane **(A,F)**, sulfate **(B,G)**, and sulfide **(C,H)**. 16S rRNA gene amplicon relative abundances for likely methane-cycling archaea **(D,I)**, and likely sulfate-reducing microbes **(E,J)**, identified based on their similarity to cultured organisms (Muyzer and Stams, 2008; Winderl et al., 2010; Waite et al., 2020; Boeuf et al., 2021; Murphy et al., 2021; Umezawa et al., 2021; Malfertheiner et al., 2022; Seidel et al., 2023). Core 1 is on top and core 2 is on the bottom. Methane error bars denote triplicate measurements on a single sample. Dashed lines on methane plots denote the depths where methane accumulates below. Legends for likely methanogens/methanotrophs and likely sulfate-reducing microbes are the same for each core.

**Figure 2:**
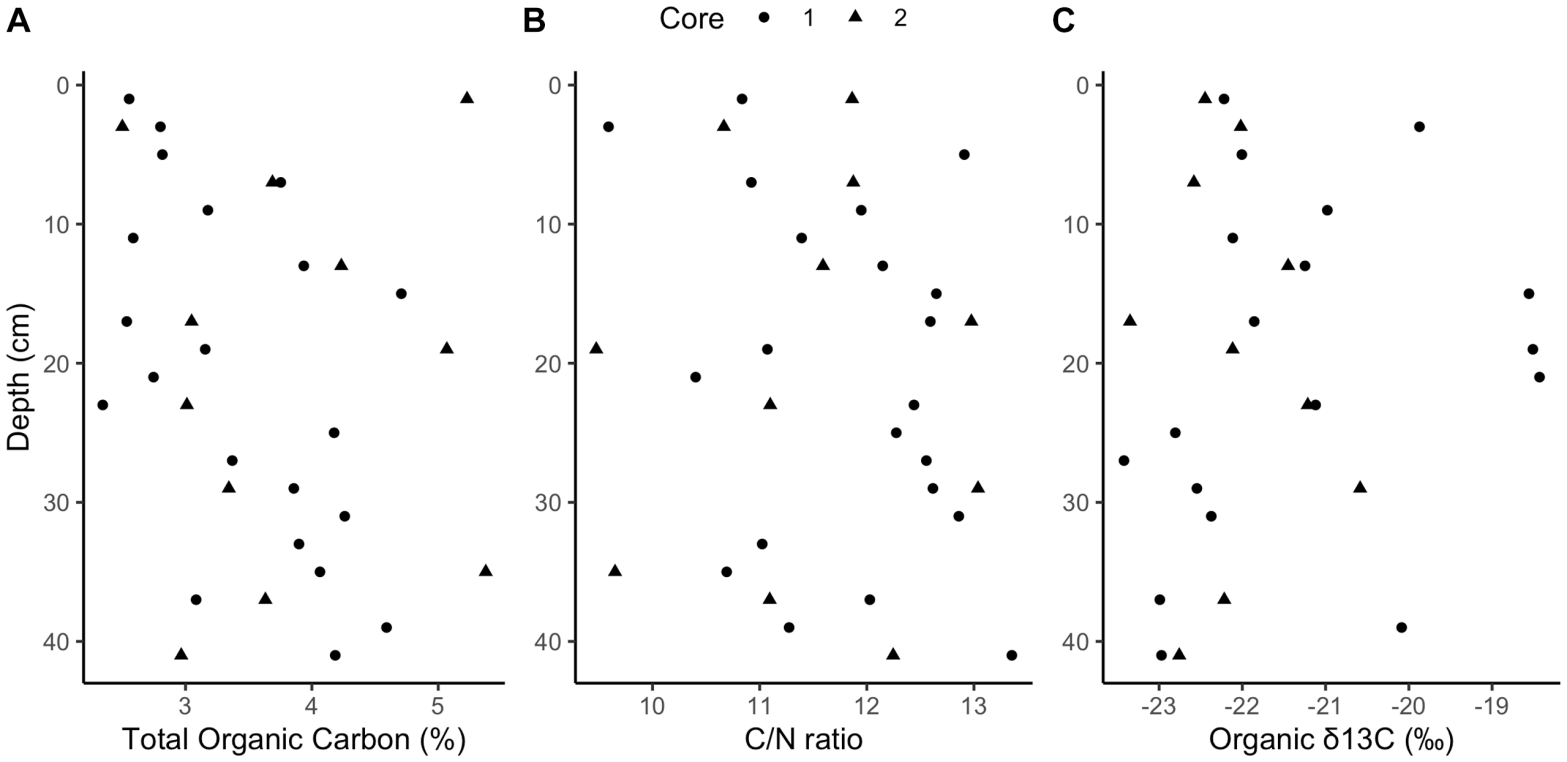
Elemental analysis. Downcore total organic carbon (TOC) concentrations (**A**, ranging from 2.4 to 5.4%), carbon to nitrogen ratios (**B**, ranging from 9 to 14), and organic stable isotope ratios (**C**, ranging from −18 to −23‰).

Methane and sulfide increase while sulfate decreases with depth in both cores (Figure 1); however, a canonical sulfate methane transition zone, where methane only begins to accumulate when sulfate is depleted, is not present in either core. Instead, methane begins accumulating while sulfate is abundant, at 6 mM and 8 mM for cores 1 and 2, respectively. Methane concentrations increase linearly rather than concave-up, e.g., with a strong methanocline, indicating a lack of net AOM, consistent with previous results from this site (Hoehler et al., 1994; Martens et al., 1998). The concavity of sulfate concentrations in core 1 and the increase in sulfide with depth in both cores demonstrate biological sulfate reduction, even as methane increases below 32 cm in core 1 and 34 cm in core 2. In core 1, methane remains less than 0.1 mM until 32 cm below sea floor (cmbsf) where it increases steadily. Methane accumulates to full saturation (1.5 mM) while sulfate concentrations are still around 5 mM at ~40 cmbsf. In core 2, methane gradually increases up to more than 0.7 mM with sulfate around 5 mM at ~40 cmbsf but never reaches full saturation within the depths sampled. The decrease in sulfate strongly correlates with the increase in sulfide between both cores (*R*^2^ = 0.75, *p-*value = 2.412e-12, DF = 36, *t*-value = −10.36, Supplementary Figure S5). Porosity mostly ranged from 70 to 85% and trended down with depth in both cores which can increase the potential aqueous methane concentrations as lower porosity increases calculated aqueous methane values (Supplementary Figure S1). DNA yields also decreased with depth (Supplementary Figure S1). Total organic carbon (TOC) concentrations ranged from 2.4 to 5.4% and C/N ratios were 9–14 (Figure 2), typical for high organic matter sites (Fuller et al., 2021). Organic matter was largely a mixture of terrestrial run-off and phytoplankton production (Buongiorno et al., 2019) with δ^13^C values −18 to −23 ‰ (Figure 2).

### 3.2. Microbial diversity of Cape Lookout Bight

**Figure 3:**
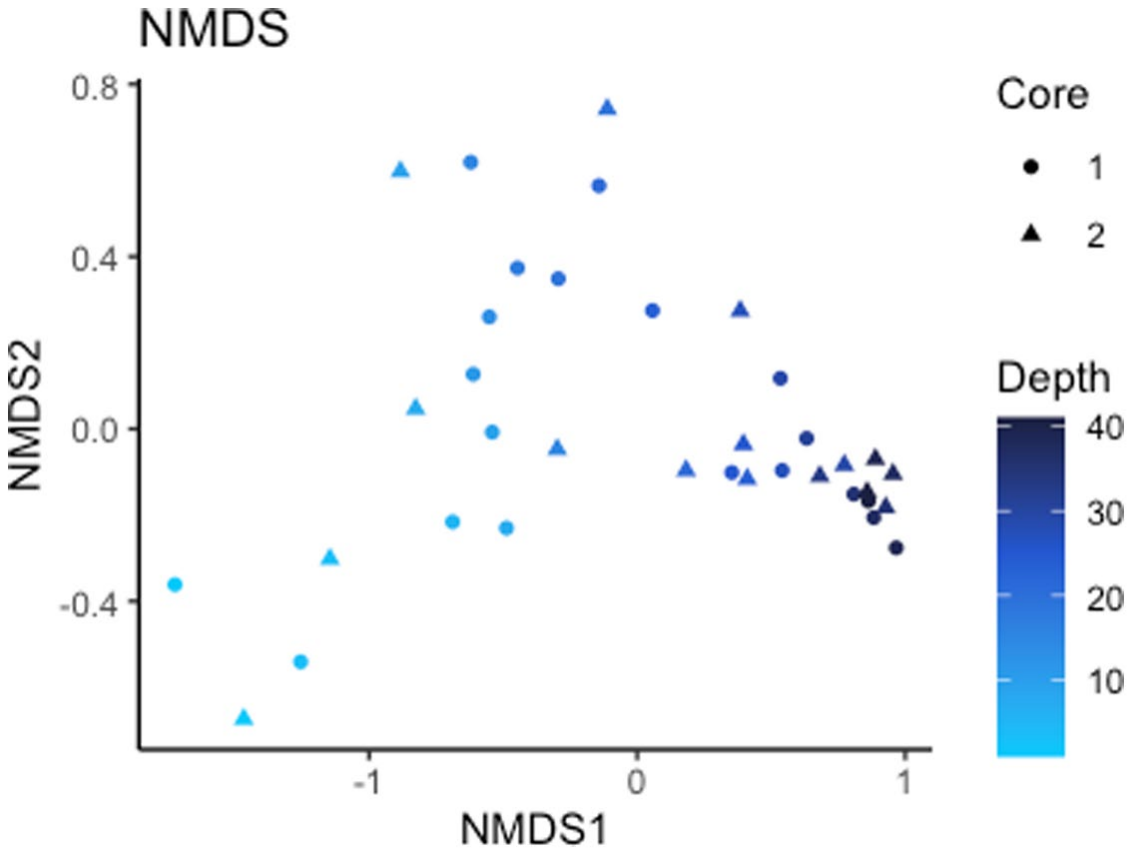
Beta diversity. NMDS of Bray-Curtis dissimilarity distances signifying depth is a driving factor of 16S rRNA gene ASV diversity.

Alpha diversity (Shannon index) of the 16S rRNA gene ASVs is 8.10 for all ASVs and 7.99 and 8.12 for core 1 and 2, respectively (Supplementary Figure S2). Non-metric multidimensional scaling (NMDS) ordination (stress <0.1) of Bray-Curtis dissimilarity distances for analyzing beta diversity show a strong correlation in taxonomy based on depth rather than which core they are from, signaling depth is a driving factor of microbial diversity present (Figure 3). Points on the NMDS plot are more distant at shallow depths while deeper microbial communities are more similar to each another, representing a convergence in communities with depth. Of the 24,987 observed ASVs (4,746,347 reads), 84.5% are Bacteria while the other 15.5% are Archaea. The total microbial distribution of phyla is shown in Supplementary Figure S3. There are 27 phyla with more than 1% abundance by ASV, and those with less than 1% are grouped into one category.

### 3.3. Composition of methane-cycling archaea and sulfate-reducing bacteria

While sulfate is still in the mM range, total methanogen abundance in sediment is 0.64% by amplicon count and 1.98% below 32 cm. The exact averaged percent between cores per depth is in Supplementary Table S2. For both cores combined, *ANME-1b* (*Ca. Methanophagales*) comprises 94% of the *ANME* archaea population by amplicon read abundance (12,533 ANME-1b reads/13,289 ANME archaea reads), which has the most abundant reads among likely methanogens/methanotrophs, at 36.1%. The rest of the community of likely methanogens/methanotrophs consists of 27.6% *Methanofastidiosales*, 20.4% *Methanosarciniales*, 16.3% *Methanomicrobiales*, 1.7% *Methanomassiliicoccales*, and <0.1% *Methanocellales*. *Methanomassiliicoccales* and *Methanofastidiosales* are known to only use methylated compounds for methane production (Nobu et al., 2016; Vanwonterghem et al., 2016; Kröninger et al., 2017; Söllinger and Urich, 2019). Cultured representatives of all other methanogens can use the hydrogenotrophic pathway except *Methanosarciniales* which is capable of using all three methanogenic pathways, hydrogenotrophic, methylotrophic, and acetoclastic (Buan, 2018). *Methanofastidiosales* reads have no family-level assignment, *Methanosarciniales* are 58.6% *Methanosaetaceae*, 34.8% *Methanosarcinaceae*, and 10% ANME 2a/2b and 2c. *Methanomicrobiales* are 62.7% *Methanomicrobiaceae*, <0.1% *Methanospirillaceae*, and the rest have no family-level assignment. There is a large abundance of reads for sulfate-reducing bacteria with *Desulfatiglans* having the most at 159,603 reads, *SEEP-SRB1* having 51,198 reads, *SVA0081* sediment group having 43,336 reads, and the rest having less than 10,000 reads. The most populous class is *Desulfobacteria* (Figure 1) and contributes the majority of class-level taxonomy identified. On average, *Desulfobacteria* makes up 6–9% of total abundance. *Methanofastidiosales* (*WSA2*) and *Methanosarciniales*, both likely capable of methylotrophic methanogenesis (Reeve et al., 1997; Nobu et al., 2016), were present in the depths with little to no methane accumulation (Figure 1). The relative abundance of *ANME-1* and *Methanomicrobiales* increase rapidly below 34 cmbsf in both cores, showing growth of the hydrogenotrophic methanogen community when methane accumulation occurs. The total relative abundance of likely sulfate-reducing bacteria remains similar throughout the cores. *SEEP-SRB1*, a sulfate-reducing bacteria with established connections to *ANME* archaea via consortia (Orphan et al., 2001), decreases with depth (*R*^2^ = 0.19, *p-*value = 0.004913, DF = 34, *t*-value = −3.009). 16S rRNA gene amplicon relative abundance of *ANME-3*, in the *Methanococcoides*, correlates with those of cultured groups of hydrogenotrophic methanogens seen during methane accumulation in Supplementary Figure S4. Likely methanogens/methanotrophs and sulfate reducers comprise a small proportion (<10%) of the total microbial population, as is commonly found in marine sediments (Colwell et al., 2008; Beulig et al., 2019; Kevorkian et al., 2022).

### 3.4. BES incubation—methanogenesis inhibition

**Figure 4:**
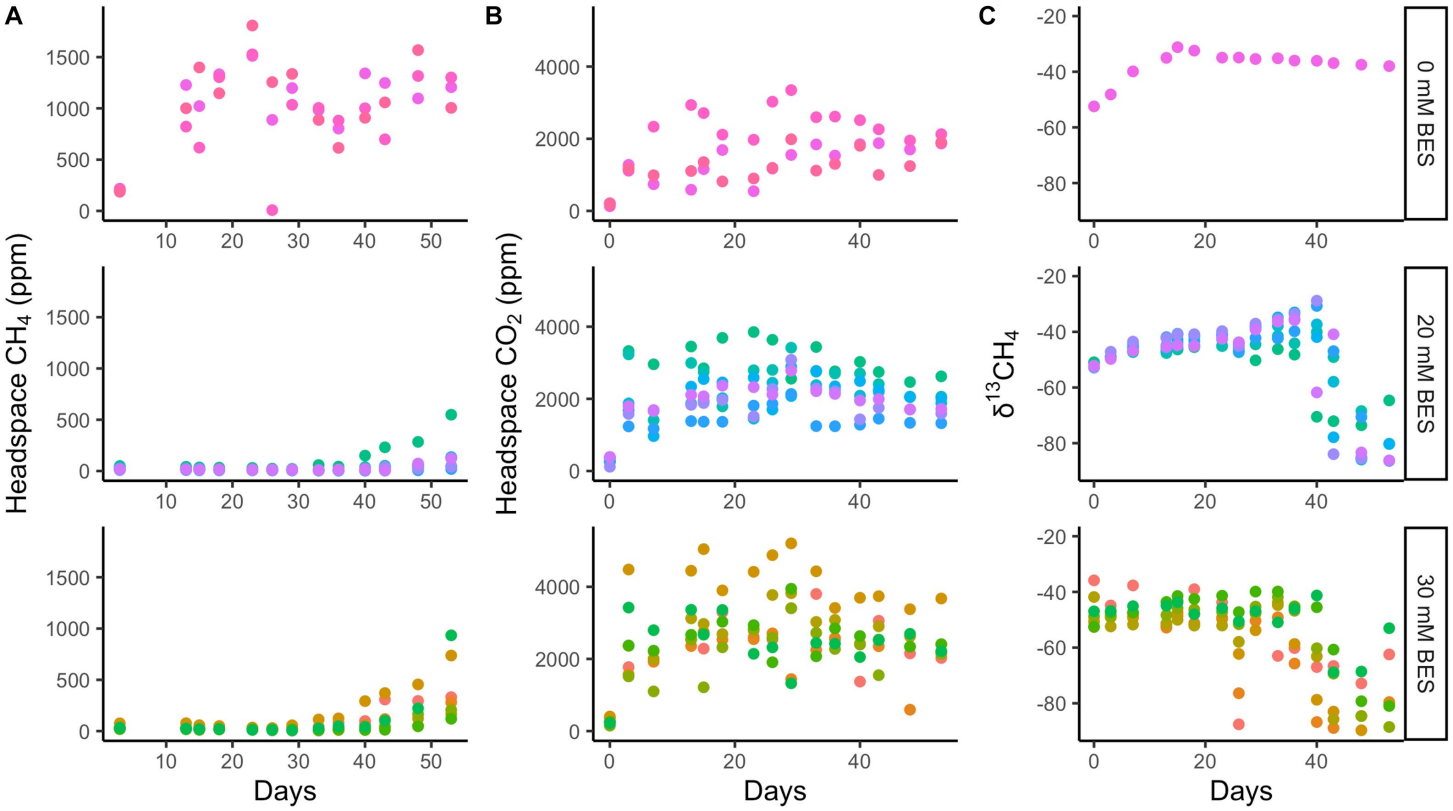
Incubation experiment 1. Bromoethanesulfonate (BES) experiment 1 results show suppression of methane production **(A)** with BES for 28 days, while CO_2_ production was enhanced with BES **(B)** and δ^13^CH_4_ shows no decrease until 40 days with BES **(C)**. The different colors represent replicate incubations and are only there to help track one replicate throughout the whole time. Rows show 0 mM, 20 mM, and 30 mM BES, top to bottom.

Whole sediment incubations with BES (both 20 mM and 30 mM BES) in incubation experiment 1 show inhibition of methane production relative to BES-free controls for at least 28 days (*p* < 0.01 for 0 mM vs. 20 mM and 30 mM treatments, two-tailed *t*-test, DF = 7; Figure 4). During this time, the BES-free controls increased headspace methane concentrations to ~1,500 ppm, while methane did not increase at all in the vials with 20 mM and 30 mM BES (Figure 4). After the first month, BES inhibition was alleviated, since the 20 mM and 30 mM BES incubations increased in headspace methane concentrations. Biological BES degradation has previously been observed in microbial fuel cells (Rago et al., 2015) and community changes have been observed in BES incubations (Whiticar, 1999), suggesting that degradation of BES over time may have decreased its ability to inhibit methanogenesis. In all incubations, δ^13^C values for methane decrease, showing ^13^C-depletion from methanogenic inputs, about 20 days after methane concentrations start to increase. This lag time could show initial methylotrophic methanogenesis which does not have such a large isotopic fractionation as hydrogenotrophic or acetoclastic methanogenesis (Conrad, 2005; Salvador et al., 2019). The CO_2_ production was similar in all three experimental conditions, suggesting that the BES-inhibition of methane production was not due to general toxicity of BES to the microbial community.

**Figure 5:**
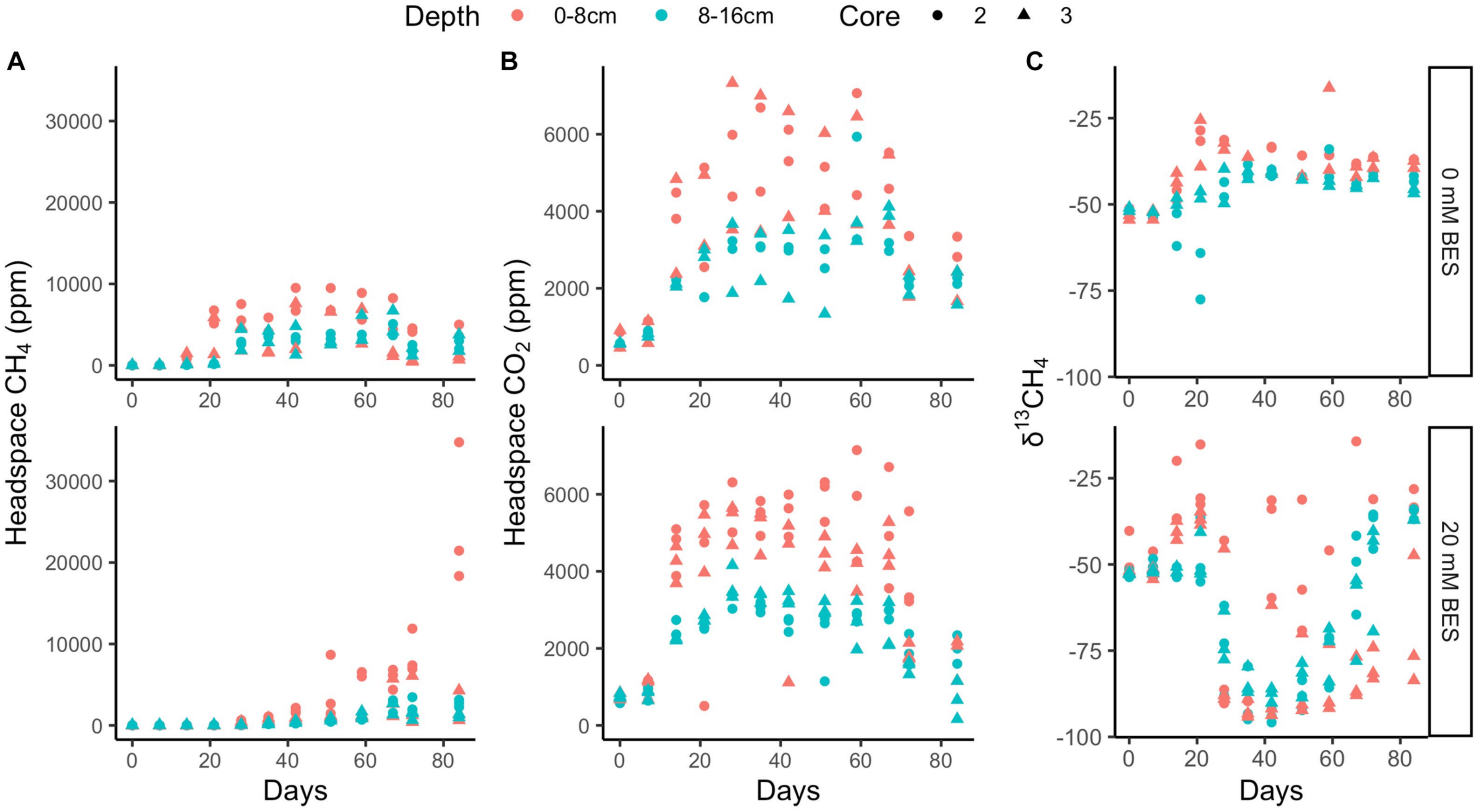
Incubation experiment 2. BES experiment 2 results show higher methane production both post-inhibition in 0–8 cm than 8–16 cm depth layers that is inhibited by 20 mM BES over 20 days and 0 mM treated incubations **(A)**, higher CO_2_ production in 0–8 cm than 8–16 cm depth layers that persists in the presence of 20 mM BES **(B)** and δ^13^CH_4_ showing methanogenesis post-inhibition at 20 days **(C)**. The different shapes represent replicate incubations.

In incubation experiment 2, methane production is inhibited in both the 0–8 cm and 8–16 cm sediment sections, since uninhibited headspace methane concentrations increased to a max of ~6,750 ppm over 35 days, while no increase occurred with 20 mM BES (*p* < 0.02 for 0 mM vs. 20 mM treatment, two-tailed *t*-test, DF = 11; Figure 5). As with experiment 1, inhibition of methane production was alleviated after this point, and δ^13^C values later decreased. The continual production of CO_2_ under all experimental conditions suggests that heterotrophy was not negatively affected by BES. Sediments from 0 to 8 cm produced more methane than sediments from 8 to 16 cm, especially after ~30 days in the BES experiments, after BES-inhibition was alleviated.

## 4. Discussion

Downcore changes in concentrations of methane and sulfate, 16S rRNA gene amplicon surveys, and incubation results suggest that methane accumulation due to a lack of net AOM occurs simultaneously to sulfate reduction while sulfate is still abundant (6–8 mM) in Cape Lookout Bight, NC.

### 4.1. Methane accumulation in the sulfate-rich sediments of Cape Lookout Bight

Previous studies have shown that the shift from net methane oxidation to net methane production occurs at the inflection point where the methane curve switches from concave up to concave down (Lloyd et al., 2011). Above this depth in sediments where net AOM occurs, the δ^13^C values of methane show net AOM by their gradual enrichment in ^13^C as methane fluxes upwards, delineating the shift from biological removal to biological production (Alperin et al., 1992). This depth often has equimolar concentrations of methane and sulfate (Iversen and Jorgensen, 1985), and methane only begins to accumulate below this depth where sulfate concentrations are very low (<0.5 mM). These trends have been observed in Eckernförde Bay (Martens et al., 1998), White Oak River estuary (Lloyd et al., 2011), Skagerrak Bay (Beulig et al., 2019), Aarhus Bay (Krause et al., 2023), Santa Barbara Basin (Komada et al., 2016), and elsewhere. In contrast to these canonical methane and sulfate profiles, our results show that methane concentrations increase below ~30 cm while sulfate concentrations are high (6 and 8 mM) and reach >0.5 mM while sulfate concentrations are still high (~5 mM) in both cores. Sulfate and sulfide concentrations profiles show that this methane accumulation occurs at depth layers where sulfate reduction also occurs. Others have reported similar profiles in CLB (Hoehler et al., 1994; Martens et al., 1998; Sturdivant and Shimizu, 2017) and Beidagang Wetland Nature Reserve (La et al., 2022). This means that in CLB, sulfate may not need to be completely depleted and sulfate reduction may not need to stop before methane accumulation begins.

The increase in methane concentrations at 32 cm in core 1 and 34 cm in core 2 coincides with an increase in MCR-containing groups of archaea, such as *Methanomicrobiales*, *Methanosarciniales*, and *ANME-1*, all capable of hydrogenotrophic methanogenesis (Buan, 2018). We attribute these methanogens as the main drivers of methane accumulation in our samples. The only other highly prevalent methanogen, *Methanofastidiosales*, does not peak after 30 cm but rather gradually increases and is predicted to be only capable of methyl-based methanogenesis (Nobu et al., 2016; Vanwonterghem et al., 2016). *Methanofastidiosales* may be a key driver of methane production in the upper cm of sediment before methane accumulation begins. This set of methane-producing archaea are commonly seen in marine sediments (Oremland and Polcin, 1982; Thauer et al., 2008; Xiao et al., 2017). When sediment from this same site was incubated previously, many of the same archaea were present (Zhuang et al., 2017). The presence of *ANME-1* in a site with no evidence for net AOM in any season provides some evidence for the ability of this clade to produce methane and drive methane accumulation in marine sediment [as suggested in Lloyd et al. (2011) and Beulig et al. (2019)], though as stated earlier there may be instances of AOM while the general process in these sediments is not methane oxidation. In estuarine sediments where AOM occurs, peaks in *ANME-1* coincide with peaks in sulfate reducers (Kevorkian et al., 2021), which is not the case in our current study.

### 4.2. Biological methane production accounts for methane accumulation in sulfate-rich sediments

The addition of 20 or 30 mM BES stops methane from accumulating for at least 20–28 days compared to BES-free incubations. As BES inhibits the MCR enzyme essential for methanogenesis and AOM (Alperin and Reeburgh, 1985; Webster et al., 2016) and the addition inhibits methane accumulation, the methane production seen in the controls must be from a biotic source for sediments up to 23 cmbsf in experiment 1 and up to 16 cmbsf in experiment 2. This shows that depths before sulfate depletion are capable of biological methane production, whether it accumulates or not. In fact, the 0–8 cmbsf section, where sulfate concentrations were higher, has greater methane production than deeper samples.

### 4.3. Methylotrophic methanogenesis throughout the sulfate-rich sediments

The potential for methane production above the depth where it begins to accumulate in sulfate-rich sediments is supported by 16S rRNA results showing that methylotrophic methanogens *Methanosarciniales* and *Methanofastidiosales* are present, which have been observed in other marine sediments (Nobu et al., 2016; Zhuang et al., 2017, 2018; Xu et al., 2021). Dominance of methylotrophic methanogens in these conditions makes sense, as it has been shown that methanogens can use methylated compounds for which sulfate reducers do not compete (Zhuang et al., 2016). While methylated compounds have been shown to be at low abundance in marine sediments, depleting rapidly with depth (Henrichs and Reeburgh, 1987; LaRowe et al., 2020), thermodynamic favorability was still observed in many studies (Martens and Klump, 1984; Canfield, 1989; Xiao et al., 2017). These methylotrophic methanogens may support the small concentrations of methane above ~30 cm in these cores. Our BES inhibition experiments support the possibility of methylotrophic methanogenesis in upper sediments since the initial methane increases were not accompanied by decreases in δ^13^CH_4_ values. It is also possible that the greater methane production in the 0–8 cm (vs. the 8–16 cm sediments) after alleviation of BES inhibition was due to demethylation of BES or its degradation products. This post-inhibition methane production was much greater than that observed in the controls.

### 4.4. Reversible hydrogenotrophic methanogenesis

**Figure 6:**
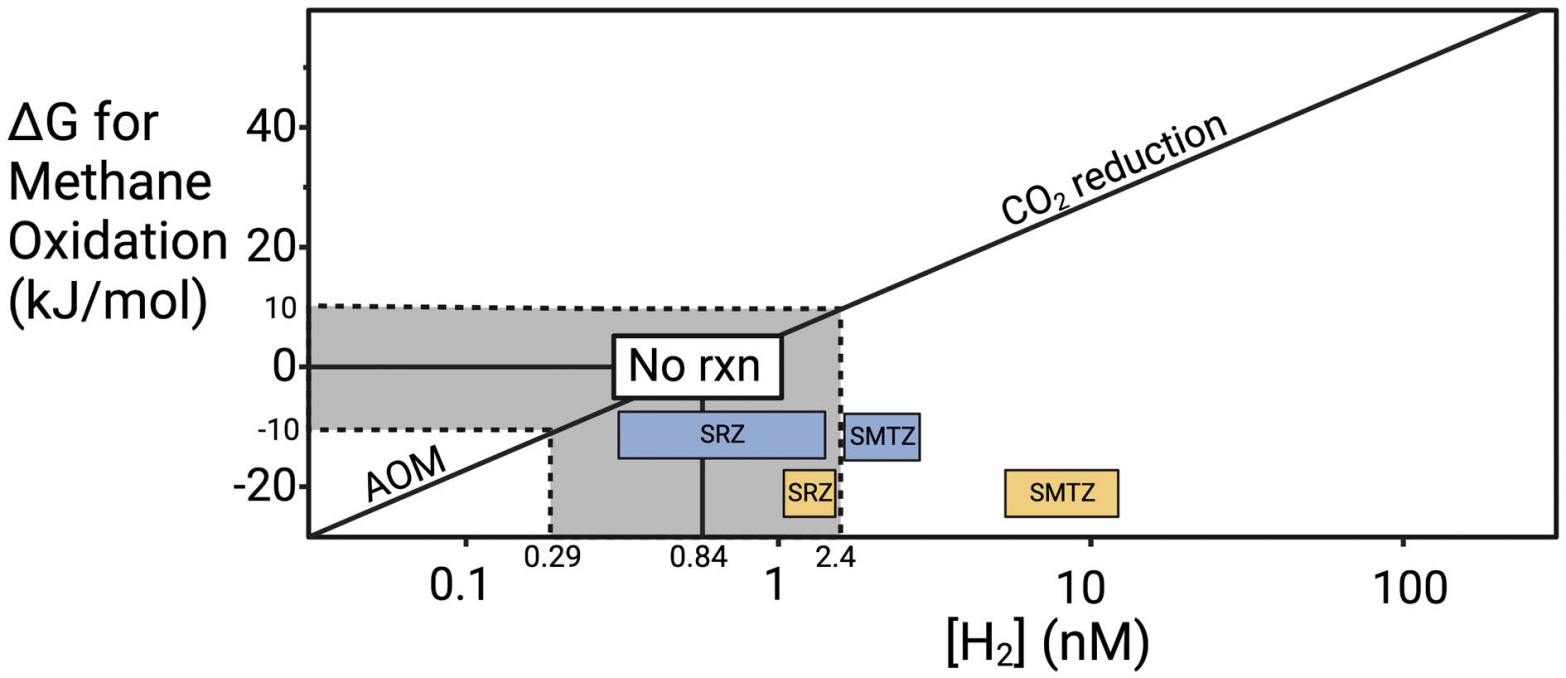
Hydrogen controls on the ΔG of anaerobic methane oxidation. Update of the “Biological energy quantum” plot from Hoehler et al. (1994), showing the ΔG values for AOM at the range of H_2_ concentrations that have been measured in marine environments (0.14–205 nM) under typical conditions at CLB (Conrad et al., 1985; Hoehler et al., 1998, 2001; Lin et al., 2012; Zhuang et al., 2017; Kevorkian et al., 2022; Lappan et al., 2023). The line modeling the ΔG values was calculated based on average CH_4_ and HCO_3_^−^ concentrations back calculated from Klump and Martens (1989) and Boehme (1993) (CH_4_ ≈ 0.53 mM and HCO_3_^−^ ≈ 48.4 mM).

Previous work provides a possible explanation for why methane accumulates before sulfate is depleted in our cores. To meet microbial maintenance requirements, a chemical reaction cannot simply be exergonic (i.e., negative ΔG value). It must exceed the energetic demand for maintenance energy, called the “biological energy quantum,” which has been measured to be ~10 kJ/mol for hydrogenotrophic methanogenesis in CLB sediments (Hoehler et al., 2001). Given that hydrogen has a stoichiometry of 4 relative to all the other products and reactants of hydrogenotrophic methanogenesis (4H_2_ + CO_2_ → CH_4_ + 2H_2_O), hydrogen largely controls the value of ΔG (Figure 6). Hydrogen has been shown to increase slightly as sulfate starts to become energetically limiting and hydrogen concentrations rise to compensate (Hoehler et al., 2001; Zhuang et al., 2017; Kevorkian et al., 2022). Many values for hydrogen fall within the “no reaction zone” where neither forward or reverse hydrogenotrophic methanogenesis meets the biological energy quantum and therefore neither supports cellular maintenance or growth. As sulfate is depleted, the increase in hydrogen, and presumably other intermediates like acetate, enables forward hydrogenotrophic methanogenesis to support cellular maintenance. Since the CLB is an area of very high organic matter lability, hydrogen concentrations may rise high enough to prevent AOM even when sulfate is present because the fermentative hydrogen flux is so high that sulfate reducers are limited by something other than hydrogen and no longer pull it to its lowest thermodynamic limit. Evidence of this increased fermentative hydrogen flux comes from the previously mentioned high rates of sedimentation and our high TOC measurements with the main drivers of fermentation likely *Chloroflexi* and *Bacteroidota* in CLB. In sites where organic matter is more recalcitrant, simultaneous sulfate reduction and methane accumulation are often not observed (Lloyd et al., 2011). In general, there is far less energy to be gained from AOM than from hydrogenotrophic methanogenesis, not because it is an inherently less energetic reaction but because there is a lower limit on hydrogen concentrations in marine sediments. The range of hydrogen concentrations that have been previously measured in marine sediments allow for a minimum ΔG of about −25 kJ/mol for AOM and a much more energetic minimum of −45 kJ/mol for hydrogenotrophic methanogenesis (Figure 6). The exact amounts depend on the concentrations of all other constituents in the reaction, but the mathematical dominance of hydrogen on ΔG allows these estimations to be useful for comparing sediments with different hydrogen concentrations. When ΔG is more negative than −10 kJ/mol, AOM through reverse hydrogenotrophic methanogenesis meets the biological energy quantum and can support cell maintenance. When ΔG is more positive than 10 kJ/mol, hydrogenotrophic methanogenesis meets the biological energy quantum and can support cell maintenance. Between −10 and +10 kJ/mol the biological energy quantum is not met and no biologically catalyzed reaction will occur. The disparity in possible energy yields for AOM vs. methanogenesis may explain why culturing archaea in low hydrogen conditions to stimulate AOM leads to slow or no replication since having enough energy to replicate would require much lower hydrogen conditions than needed to just meet the BEQ. If AOM occurred through direct interspecies electron transfer at CLB sediments, then it should prevent the accumulation of methane when sulfate is present. Since that does not occur, reversible hydrogenotrophic methanogenesis is a more likely explanation.

This proposed mechanism relies on excess hydrogen from high fermentation rates. Two known common phyla of fermenters are present and potentially producing much of the available hydrogen by fermenting the relatively high concentrations of organic matter (Kendall et al., 2007). *Chloroflexi* and *Bacteroidota* (also known as *Bacteroidetes*) are roughly a quarter of the population by amplicon count (Supplementary Figure S3). The high TOC seen in Figure 2 and the previously measured high anaerobic remineralization rates (Martens et al., 1998; Sprenger et al., 2000; Underwood et al., 2016; Kevorkian et al., 2018) suggest high levels of fermentation from these phyla and potentially others, which would yield hydrogen to help power methanogenesis in the presence of sulfate.

## 5. Conclusion

We present 16S rRNA data for microbial communities in duplicate cores of organic-rich sediments of Cape Lookout Bight, NC, where methane accumulates in the presence of 6–8 mM sulfate while sulfate reduction also occurs. This lack of AOM during sulfate reduction may be the result of the highly labile organic matter allowing for fermentation to supply excess hydrogen that sulfate-reducing bacteria do not completely use. Methane accumulation below ~30 cm in the sulfate-rich sediments is accompanied by increases in *ANME-1*, *Methanosarciniales* and *Methanomicrobiales*, but not sulfate-reducing bacteria. *Methanofastidiosales* is present throughout the sediments and may account for methylotrophic methanogenesis which results in little methane accumulation. Methane production throughout these sediments is biotic since it is inhibited by BES. These results support the theory of reversible hydrogenotrophic methanogenesis as the main driver for AOM and methanogenesis in coastal marine sediments since the presence of sulfate alone is insufficient to prevent methane accumulation.

## Data availability statement

The datasets presented in this study can be found in online repositories. The names of the repository/repositories and accession number(s) can be found at: https://www.ncbi.nlm.nih.gov/, PRJNA949635.

## Author contributions

GC: Conceptualization, Data curation, Formal analysis, Investigation, Methodology, Resources, Software, Visualization, Writing – original draft, Writing – review & editing. PD: Conceptualization, Investigation, Methodology, Resources, Writing – review & editing. RP: Investigation, Resources, Software, Visualization, Writing – review & editing. JB: Investigation, Resources, Software, Writing – review & editing. KL: Conceptualization, Funding acquisition, Methodology, Project administration, Resources, Supervision, Writing – review & editing.
