# Supplementary material for: Biological methane production and accumulation under sulfate-rich conditions at Cape Lookout Bight, NC
Source: Front Microbiol. 2023 Oct 6;14:1268361. doi: 10.3389/fmicb.2023.1268361 (PMC10587565; doi:10.3389/fmicb.2023.1268361)
Supplement: Supplementary file 1 [file Data_Sheet_1.docx]

Supplementary Material

# Supplementary Figures and Tables

## Supplementary Tables

**Supplementary Table 1.** Reads from 16S rRNA assay for each sample and the blank, showing input before any processing, cleaned is after filtering, denoising, merging, and removing chimeras based on the dada2 pipeline’s recommendations, and the after_decontaminaton is after removal of list of common contaminants from Sheik et al. 2018 Frontiers in Microbiology (included in code).

| sample | input | cleaned | after_decontamination |
| --- | --- | --- | --- |
| 0-2-CLB1 | 172897 | 135454 | 122555 |
| 0-2-CLB2 | 228369 | 175425 | 164902 |
| 10-12-CLB1 | 179850 | 134221 | 131231 |
| 10-12-CLB2 | 1880 | 753 | removed |
| 12-14-CLB1 | 164057 | 119828 | 116741 |
| 12-14-CLB2 | 514 | 13 | removed |
| 14-16-CLB1 | 155369 | 116058 | 113320 |
| 14-16-CLB2 | 215488 | 162689 | 155818 |
| 16-18-CLB1 | 103281 | 74402 | 70881 |
| 16-18-CLB2 | 11945 | 6811 | removed |
| 18-20-CLB1 | 135918 | 96692 | 92767 |
| 18-20-CLB2 | 210811 | 155642 | 152551 |
| 2-4-CLB1 | 181261 | 134644 | 124121 |
| 2-4-CLB2 | 228799 | 173386 | 160017 |
| 20-22-CLB1 | 120500 | 86706 | 83845 |
| 20-22-CLB2 | 206100 | 160097 | 157177 |
| 22-24-CLB1 | 118369 | 83548 | 82257 |
| 22-24-CLB2 | 215920 | 169984 | 168467 |
| 24-26-CLB1 | 139894 | 107496 | 106029 |
| 24-26-CLB2 | 219491 | 170301 | 168054 |
| 26-28-CLB1 | 134319 | 101732 | 100496 |
| 26-28-CLB2 | 172175 | 129902 | 128354 |
| 28-30-CLB1 | 127078 | 95161 | 93831 |
| 28-30-CLB2 | 165577 | 121602 | 120625 |
| 30-32-CLB1 | 132685 | 100828 | 99991 |
| 30-32-CLB2 | 5062 | 2950 | removed |
| 32-34-CLB1 | 18675 | 12078 | removed |
| 32-34-CLB2 | 201991 | 158973 | 157545 |
| 34-36-CLB1 | 217684 | 171520 | 170326 |
| 34-36-CLB2 | 176682 | 138941 | 137873 |
| 36-38-CLB1 | 202296 | 158450 | 157492 |
| 36-38-CLB2 | 160796 | 125497 | 124574 |
| 38-40-CLB1 | 150367 | 106829 | 106095 |
| 38-40-CLB2 | 203810 | 160884 | 159703 |
| 4-6-CLB1 | 166834 | 124147 | 120214 |
| 4-6-CLB2 | 13981 | 8130 | removed |
| 40-42-CLB1 | 239494 | 189348 | 187968 |
| 40-42-CLB2 | 211100 | 166614 | 165731 |
| 6-8-CLB1 | 150754 | 111640 | 109426 |
| 6-8-CLB2 | 201072 | 149262 | 143962 |
| 8-10-CLB1 | 193599 | 145234 | 142559 |
| 8-10-CLB2 | 205177 | 153750 | 148849 |
| JBpcrBlank | 1000 | 129 | removed |

**Supplementary Table 2.** Average relative abundance of methanogens discussed in main text per depth.

| Depth | Percent methanogen |
| --- | --- |
| 1 | 0.06089601 |
| 3 | 0.13276376 |
| 5 | 0.12477748 |
| 7 | 0.15293556 |
| 9 | 0.09674761 |
| 11 | 0.12954256 |
| 13 | 0.11135762 |
| 15 | 0.17331997 |
| 17 | 0.18199518 |
| 19 | 0.14231343 |
| 21 | 0.24528807 |
| 23 | 0.30106473 |
| 25 | 0.29233275 |
| 27 | 0.31005718 |
| 29 | 0.44990033 |
| 31 | 0.57105139 |
| 33 | 0.50969564 |
| 35 | 1.49193036 |
| 37 | 2.63166355 |
| 39 | 2.45881971 |
| 41 | 2.81641253 |

**Supplementary Table 3.** Includes all imported R packages and versions included in all code.

| Package | Version |
| --- | --- |
| abind | 1.4-5 |
| ade4 | 1.7-22 |
| ape | 5.7 |
| askpass | 1.1 |
| assertthat | 0.2.1 |
| backports | 1.4.1 |
| base64enc | 0.1-3 |
| base64url | 1.4 |
| bayesm | 3.1-5 |
| BH | 1.81.0-1 |
| Biobase | 2.54.0 |
| BiocGenerics | 0.40.0 |
| BiocManager | 1.30.20 |
| BiocParallel | 1.28.3 |
| BiocVersion | 3.14.0 |
| biomformat | 1.22.0 |
| Biostrings | 2.62.0 |
| bit | 4.0.5 |
| bit64 | 4.0.5 |
| bitops | 1.0-7 |
| blob | 1.2.3 |
| brew | 1.0-8 |
| brio | 1.1.3 |
| broom | 1.0.3 |
| bslib | 0.4.2 |
| cachem | 1.0.7 |
| callr | 3.7.3 |
| car | 3.1-1 |
| carData | 3.0-5 |
| cellranger | 1.1.0 |
| checkmate | 2.1.0 |
| CHNOSZ | 2.0.0 |
| circlize | 0.4.15 |
| classInt | 0.4-9 |
| cli | 3.6.0 |
| clipr | 0.8.0 |
| coda | 0.19-4 |
| coda.base | 0.5.4.3 |
| colorspace | 2.1-0 |
| commonmark | 1.9.0 |
| compositions | 2.0-5 |
| conflicted | 1.2.0 |
| corrplot | 0.92 |
| cowplot | 1.1.1 |
| cpp11 | 0.4.3 |
| crayon | 1.5.2 |
| credentials | 1.3.2 |
| crosstalk | 1.2.0 |
| curl | 5.0.0 |
| dada2 | 1.22.0 |
| data.table | 1.14.8 |
| DBI | 1.1.3 |
| dbplyr | 2.3.1 |
| DECIPHER | 2.22.0 |
| DelayedArray | 0.20.0 |
| deldir | 1.0-6 |
| dendextend | 1.16.0 |
| DEoptimR | 1.0-11 |
| desc | 1.4.2 |
| devtools | 2.4.5 |
| diffobj | 0.3.5 |
| digest | 0.6.31 |
| doParallel | 1.0.17 |
| doRNG | 1.8.6 |
| downlit | 0.4.2 |
| dplyr | 1.1.0 |
| DT | 0.27 |
| dtplyr | 1.3.0 |
| e1071 | 1.7-13 |
| ellipse | 0.4.3 |
| ellipsis | 0.3.2 |
| emmeans | 1.8.4-1 |
| estimability | 1.4.1 |
| evaluate | 0.2 |
| factoextra | 1.0.7 |
| FactoMineR | 2.7 |
| fansi | 1.0.4 |
| farver | 2.1.1 |
| fastcluster | 1.2.3 |
| fastmap | 1.1.1 |
| flashClust | 1.01-2 |
| fontawesome | 0.5.0 |
| forcats | 1.0.0 |
| foreach | 1.5.2 |
| formatR | 1.14 |
| Formula | 1.2-5 |
| fs | 1.6.1 |
| futile.logger | 1.4.3 |
| futile.options | 1.0.1 |
| gargle | 1.3.0 |
| generics | 0.1.3 |
| GenomeInfoDb | 1.30.1 |
| GenomeInfoDbData | 1.2.7 |
| GenomicAlignments | 1.30.0 |
| GenomicRanges | 1.46.1 |
| gert | 1.9.2 |
| ggalluvial | 0.12.5 |
| ggforce | 0.4.1 |
| gghalves | 0.1.4 |
| ggplot2 | 3.4.1 |
| ggpubr | 0.6.0 |
| ggraph | 2.1.0 |
| ggrepel | 0.9.3 |
| ggsci | 2.9 |
| ggsignif | 0.6.4 |
| ggtern | 3.4.1 |
| ggthemes | 4.2.4 |
| gh | 1.4.0 |
| gitcreds | 0.1.2 |
| GlobalOptions | 0.1.2 |
| glue | 1.6.2 |
| googledrive | 2.0.0 |
| googlesheets4 | 1.0.1 |
| graph | 1.72.0 |
| graphlayouts | 0.8.4 |
| gridExtra | 2.3 |
| gtable | 0.3.1 |
| haven | 2.5.2 |
| heatmap3 | 1.1.9 |
| hexbin | 1.28.2 |
| highr | 0.1 |
| Hmisc | 4.7-2 |
| hms | 1.1.2 |
| htmlTable | 2.4.1 |
| htmltools | 0.5.4 |
| htmlwidgets | 1.6.1 |
| httpuv | 1.6.9 |
| httr | 1.4.5 |
| httr2 | 0.2.2 |
| hwriter | 1.3.2.1 |
| ids | 1.0.1 |
| igraph | 1.4.1 |
| ini | 0.3.1 |
| intergraph | 2.0-2 |
| interp | 1.1-3 |
| IRanges | 2.28.0 |
| IRdisplay | 1.1 |
| IRkernel | 1.3.2 |
| isoband | 0.2.7 |
| iterators | 1.0.14 |
| itertools | 0.1-3 |
| jpeg | 0.1-10 |
| jquerylib | 0.1.4 |
| jsonlite | 1.8.4 |
| knitr | 1.42 |
| labeling | 0.4.2 |
| lambda.r | 1.2.4 |
| later | 1.3.0 |
| latex2exp | 0.9.6 |
| latticeExtra | 0.6-30 |
| lazyeval | 0.2.2 |
| leaps | 3.1 |
| lemon | 0.4.6 |
| lifecycle | 1.0.3 |
| lme4 | 1.1-31 |
| lubridate | 1.9.2 |
| magrittr | 2.0.3 |
| maptools | 1.1-6 |
| markdown | 1.7 |
| MatrixGenerics | 1.6.0 |
| MatrixModels | 0.5-1 |
| matrixStats | 0.63.0 |
| memoise | 2.0.1 |
| metagMisc | 0.0.4 |
| microbiome | 1.16.0 |
| microbiomeutilities | 1.00.16 |
| mime | 0.12 |
| miniUI | 0.1.1.1 |
| minqa | 1.2.5 |
| missForest | 1.5 |
| modelr | 0.1.10 |
| multcompView | 0.1-8 |
| multtest | 2.50.0 |
| munsell | 0.5.0 |
| mvtnorm | 1.1-3 |
| network | 1.18.1 |
| nloptr | 2.0.3 |
| numDeriv | 2016.8-1.1 |
| openssl | 2.0.5 |
| pbdZMQ | 0.3-9 |
| pbkrtest | 0.5.2 |
| permute | 0.9-7 |
| pheatmap | 1.0.12 |
| phyloseq | 1.38.0 |
| phylosmith | 1.0.6 |
| pillar | 1.8.1 |
| pixmap | 0.4-12 |
| pkgbuild | 1.4.0 |
| pkgconfig | 2.0.3 |
| pkgdown | 2.0.7 |
| pkgload | 1.3.2 |
| plogr | 0.2.0 |
| plyr | 1.8.8 |
| png | 0.1-8 |
| polyclip | 1.10-4 |
| polynom | 1.4-1 |
| praise | 1.0.0 |
| prettyunits | 1.1.1 |
| processx | 3.8.0 |
| profvis | 0.3.7 |
| progress | 1.2.2 |
| promises | 1.2.0.1 |
| propr | 4.2.6 |
| proto | 1.0.0 |
| proxy | 0.4-27 |
| ps | 1.7.2 |
| purrr | 1.0.1 |
| quantreg | 5.94 |
| R6 | 2.5.1 |
| ragg | 1.2.5 |
| randomForest | 4.7-1.1 |
| ranger | 0.14.1 |
| rappdirs | 0.3.3 |
| Rborist | 0.3-2 |
| rcmdcheck | 1.4.0 |
| RColorBrewer | 1.1-3 |
| Rcpp | 1.0.10 |
| RcppArmadillo | 0.12.0.1.0 |
| RcppEigen | 0.3.3.9.3 |
| RcppParallel | 5.1.7 |
| RcppProgress | 0.4.2 |
| RCurl | 1.98-1.10 |
| RCy3 | 2.14.2 |
| readr | 2.1.4 |
| readxl | 1.4.2 |
| rematch | 1.0.1 |
| rematch2 | 2.1.2 |
| remotes | 2.4.2 |
| repr | 1.1.6 |
| reprex | 2.0.2 |
| reshape2 | 1.4.4 |
| rhdf5 | 2.38.1 |
| rhdf5filters | 1.6.0 |
| Rhdf5lib | 1.16.0 |
| Rhtslib | 1.26.0 |
| rioja | 1.0-5 |
| rJava | 1.0-6 |
| RJSONIO | 1.3-1.8 |
| rlang | 1.0.6 |
| rmarkdown | 2.2 |
| rngtools | 1.5.2 |
| robustbase | 0.95-0 |
| roxygen2 | 7.2.3 |
| rprojroot | 2.0.3 |
| Rsamtools | 2.10.0 |
| RSQLite | 2.3.0 |
| rstatix | 0.7.2 |
| rstudioapi | 0.14 |
| Rtsne | 0.16 |
| rversions | 2.1.2 |
| rvest | 1.0.3 |
| s2 | 1.1.2 |
| S4Vectors | 0.32.4 |
| sass | 0.4.5 |
| scales | 1.2.1 |
| scatterplot3d | 0.3-42 |
| selectr | 0.4-2 |
| sessioninfo | 1.2.2 |
| sf | 1.0-9 |
| shape | 1.4.6 |
| shiny | 1.7.4 |
| ShortRead | 1.52.0 |
| snow | 0.4-4 |
| sourcetools | 0.1.7-1 |
| sp | 1.6-0 |
| SparseM | 1.81 |
| statnet.common | 4.8.0 |
| stringi | 1.7.12 |
| stringr | 1.5.0 |
| SummarizedExperiment | 1.24.0 |
| sys | 3.4.1 |
| systemfonts | 1.0.4 |
| tensorA | 0.36.2 |
| testthat | 3.1.6 |
| textshaping | 0.3.6 |
| threejs | 0.3.3 |
| tibble | 3.1.8 |
| tidygraph | 1.2.3 |
| tidyr | 1.3.0 |
| tidyselect | 1.2.0 |
| tidyverse | 2.0.0 |
| timechange | 0.2.0 |
| tinytex | 0.44 |
| tweenr | 2.0.2 |
| tzdb | 0.3.0 |
| uchardet | 1.1.1 |
| units | 0.8-1 |
| urlchecker | 1.0.1 |
| usethis | 2.1.6 |
| utf8 | 1.2.3 |
| uuid | 1.1-0 |
| vctrs | 0.5.2 |
| vegan | 2.6-4 |
| viridis | 0.6.2 |
| viridisLite | 0.4.1 |
| vroom | 1.6.1 |
| VSURF | 1.1.0 |
| waldo | 0.4.0 |
| whisker | 0.4.1 |
| withr | 2.5.0 |
| wk | 0.7.1 |
| writexl | 1.4.2 |
| xfun | 0.39 |
| xlsx | 0.6.5 |
| xlsxjars | 0.6.1 |
| XML | 3.99-0.13 |
| xml2 | 1.3.3 |
| xopen | 1.0.0 |
| xtable | 1.8-4 |
| XVector | 0.34.0 |
| yaml | 2.3.7 |
| zip | 2.2.2 |
| zlibbioc | 1.40.0 |

**Supplementary Table 4.** All downcore geochemical data used besides elemental analysis. Out of range means out of the instrument’s detection range.

| Depth | Sulfide (mM) | Porosity SD | Porosity | Methane (mM) | Core | Methane SD | Sulfate (mM) | DNA (ng/mL) |
| --- | --- | --- | --- | --- | --- | --- | --- | --- |
| 1 | 0.24271357 | 0.033361394 | 0.80417911 | 0.090472861 | 1 | 0.0057886 | 21.7405161 | Out of range |
| 3 | 1.13467337 | 0.004166521 | 0.81153346 | 0.018234502 | 1 | 0.01184366 | 16.5185264 | Out of range |
| 5 | 1.18994975 | 0.020416764 | 0.76467729 | 0.029027753 | 1 | 0.00241898 | 14.5567989 | Out of range |
| 7 | 1.8758794 | 0.003286191 | 0.76255761 | 0.015362788 | 1 | 0.00140048 | 11.9006144 | Out of range |
| 9 | 1.67236181 | 0.023427431 | 0.71833177 | 0.527884787 | 1 | 0.01287524 | 11.3856876 | 56 |
| 11 | 2.10954774 | 0.008880967 | 0.73044556 | 0.025070194 | 1 | 0.00043861 | 11.3782698 | 52 |
| 13 | 1.9839196 | 0.003451074 | 0.78377868 | 0.01416016 | 1 | 0.00236003 | 9.99053882 | 59 |
| 15 | 2.24522613 | 0.001596108 | 0.78297861 | 0.271680398 | 1 | 0.05181232 | 9.76932284 | 54 |
| 17 | 2.31055276 | 0.039601509 | 0.71996898 | 0.033399497 | 1 | 0.00513838 | 9.6899108 | 33.7 |
| 19 | 2.79284925 | 0.044951112 | 0.75383863 | 0.030263032 | 1 | 0.00141668 | 7.85883772 | 51 |
| 21 | 2.32562814 | 0.004995727 | 0.75077587 | 0.044347849 | 1 | 0.00426737 | 4.63261582 | 46.5 |
| 23 | 2.73009799 | 0.01534627 | 0.58918199 | 0.118254611 | 1 | 0.02042688 |  | 51 |
| 25 | 2.79284925 | 0.010485042 | 0.7028078 | 0.050883916 | 1 | 0.00402033 | 6.89469828 | 56 |
| 27 | 3.5165804 | 0.000686239 | 0.71933982 | 0.078000323 | 1 | 0.00647131 | 7.45759079 | 8.73 |
| 29 | 2.97273618 | 0.035773075 | 0.73310179 | 0.120270984 | 1 | 0.02767828 | 6.12829253 | 18.7 |
| 31 | 2.94345226 | 0.022451207 | 0.71984652 | 0.022270119 | 1 | 0.00148358 | 5.49640939 | 56 |
| 33 | 2.7091809 | 0.000607059 | 0.73659399 | 0.532375488 | 1 | 0.0205551 | 4.84759716 | 0.767 |
| 35 | 3.67555025 | 0.016592037 | 0.73245733 | 0.353554254 | 1 | 0.03471834 | 4.3297579 | 42.1 |
| 37 | 2.81794975 | 0.010765993 | 0.71404147 | 0.902364416 | 1 | 0.01724853 |  | 47.8 |
| 39 | 3.40362814 | 0.017754194 | 0.77155312 | 1.249056993 | 1 | 0.03690785 | 3.79744698 | 54 |
| 41 | 3.24884171 | 0.002872219 | 0.72949706 | 1.527297746 | 1 | 0.21801059 | 3.03035861 | 50 |
| 1 | 0.42110553 | 0.017283127 | 0.84095953 | 0.04985663 | 2 | 0.00507966 | 19.6970556 | 51 |
| 3 | 1.05175879 | 0.030529193 | 0.76884773 | 0.063354266 | 2 | 0.01837505 | 18.9837126 | 38.5 |
| 5 | 1.70251256 | 0.036214492 | 0.75277087 | 0.149891634 | 2 | 0.00650125 | 14.2388778 | 0.719 |
| 7 | 2.02663317 | 0.031004147 | 0.80548878 | 0.207443057 | 2 | 0.03217717 | 13.076049 | 56 |
| 9 | 2.02160804 | 0.043877157 | 0.80143414 | 0.199260415 | 2 | 0.02144528 | 14.5933876 | 26.6 |
| 11 | 2.13969849 | 0.036804121 | 0.73237273 | 0.12544207 | 2 | 0.0211816 | 14.5822836 | 0 |
| 13 | 2.18743719 | 0.008140418 | 0.75062547 | 0.078034997 | 2 | 0.00376422 | 14.0696323 | 55 |
| 15 | 1.96633166 | 0.013844052 | 0.76137965 | 0.309350773 | 2 | 0.0212259 | 14.9443479 | 33.5 |
| 17 | 2.25527638 | 0.018714162 | 0.73473857 | 0.208956371 | 2 | 0.02555031 | 11.3103713 | Out of range |
| 19 | 2.52092714 | 0.032676773 | 0.75087419 | 0.271800297 | 2 | 0.02006361 | 9.42796487 | 51 |
| 21 | 2.40379146 | 0.002366032 | 0.73699023 | 0.348869844 | 2 | 0.02850647 | 11.50542 | 13.4 |
| 23 | 1.88923116 | 0.007925381 | 0.74777066 | 0.033806837 | 2 | 0.00142817 | 12.084559 | 48.6 |
| 25 | 2.01055025 | 0.02397499 | 0.76966699 | 0.177042902 | 2 | 0.01942562 | 3.76026668 | 46.9 |
| 27 | 2.54184422 | 0.016624306 | 0.81711951 | 0.122241464 | 2 | 0.00784179 |  | 10.7 |
| 29 | 2.73428141 | 0.031637605 | 0.7061929 | 0.411231776 | 2 | 0.01593264 |  | 58 |
| 31 | 2.4874598 | 0.024029374 | 0.7384301 | 0.405803684 | 2 | 0.01252481 | 10.6300674 | 0.239 |
| 33 | 2.40797487 | 0.001885294 | 0.73019493 | 0.398558701 | 2 | 0.01197149 | 6.75152908 | 6.9 |
| 35 | 2.96018593 | 0.009644916 | 0.74473868 | 0.563809397 | 2 | 0.02628545 | 4.5718622 | 39 |
| 37 | 2.45399246 | 0.007859456 | 0.71887122 | 0.580666377 | 2 | 0.19546082 | 8.76340675 | 34.8 |
| 39 | 2.45817588 | 0.034095916 | 0.72560838 | 0.094321304 | 2 | 0.01111181 | 9.89324201 | 3.31 |
| 41 | 2.75938191 | 0.024584926 | 0.69797002 | 0.722612786 | 2 | 0.13307263 | 7.5237599 | 44.4 |

**Supplementary Table 5.** All elemental analysis data. Blanks mean no quality data was produced during measurement and was not ran again.

| Depth | Core | d13C Total | d13C Inorganic | d13C Organic | C/N Nontreated | C/N Treated |
| --- | --- | --- | --- | --- | --- | --- |
| 1 | 1 | -18.373 | 3.847 | -22.22 | 11.2379227 | 10.8358082 |
| 3 | 1 | -17.637 | 2.235 | -19.872 | 11.4590273 | 9.5892521 |
| 5 | 1 | -17.137 | 4.869 | -22.006 | 11.25789 | 12.9101742 |
| 7 | 1 | -16.104 |  |  | 12.4734043 | 10.922316 |
| 9 | 1 | -17.321 | 3.658 | -20.979 | 11.7403767 | 11.9481008 |
| 11 | 1 | -17.58 | 4.535 | -22.115 | 11.2085944 | 11.3920705 |
| 13 | 1 | -17.178 | 4.069 | -21.247 | 11.6922487 | 12.1481481 |
| 15 | 1 | -17.162 | 1.395 | -18.557 | 12.1125675 | 12.6491134 |
| 17 | 1 | -14.128 | 7.729 | -21.857 | 14.9156627 | 12.5931446 |
| 19 | 1 | -16.428 | 2.082 | -18.51 | 12.3840445 | 11.0721302 |
| 21 | 1 | -16.671 | 1.759 | -18.43 | 11.5486348 | 10.4021661 |
| 23 | 1 | -14.845 | 6.275 | -21.12 | 13.7153197 | 12.4403183 |
| 25 | 1 | -15.559 | 7.246 | -22.805 | 13.3067332 | 12.2751323 |
| 27 | 1 | -16.123 | 7.299 | -23.422 | 12.7758319 | 12.5558867 |
| 29 | 1 | -16.729 | 5.817 | -22.546 | 11.8447489 | 12.6169447 |
| 31 | 1 | -15.788 | 6.585 | -22.373 | 12.1023857 | 12.8584365 |
| 33 | 1 | -17.091 |  |  | 11.9353567 | 11.0237557 |
| 35 | 1 | -16.337 |  |  | 12.4042951 | 10.6919232 |
| 37 | 1 | -16.81 | 6.181 | -22.991 | 11.8691589 | 12.0280811 |
| 39 | 1 | -15.815 | 4.271 | -20.086 | 12.9976762 | 11.274558 |
| 41 | 1 | -16.525 | 6.446 | -22.971 | 12.2418358 | 13.3535418 |
| 1 | 2 | -16.985 | 5.463 | -22.448 | 11.8609023 | 11.8629453 |
| 3 | 2 | -17.186 | 4.833 | -22.019 | 11.6878711 | 10.665529 |
| 5 | 2 |  |  |  |  |  |
| 7 | 2 | -16.832 | 5.75 | -22.582 | 12.7344134 | 11.8737927 |
| 9 | 2 |  |  |  |  |  |
| 11 | 2 |  |  |  |  |  |
| 13 | 2 | -16.286 | 5.164 | -21.45 | 12.8183054 | 11.5909091 |
| 15 | 2 |  |  |  |  |  |
| 17 | 2 | -17.448 | 5.901 | -23.349 | 14.1825558 | 12.9757344 |
| 19 | 2 | -16.798 | 5.319 | -22.117 | 11.7827796 | 9.47466816 |
| 21 | 2 |  |  |  |  |  |
| 23 | 2 | -16.258 | 4.956 | -21.214 | 12.754734 | 11.098415 |
| 25 | 2 |  |  |  |  |  |
| 27 | 2 |  |  |  |  |  |
| 29 | 2 | -15.643 | 4.942 | -20.585 | 12.5182927 | 13.0382215 |
| 31 | 2 |  |  |  |  |  |
| 33 | 2 |  |  |  |  |  |
| 35 | 2 | -15.965 |  |  | 12.8751821 | 9.64997307 |
| 37 | 2 | -17.634 | 4.583 | -22.217 | 11.9604317 | 11.0934637 |
| 39 | 2 |  |  |  |  |  |
| 41 | 2 | -15.748 | 7.01 | -22.758 | 13.3438685 | 12.2460776 |

## Supplementary Files

**Supplementary File 1.** All BES incubation data.

Experiment 1 - Methane

| Sample | BES | 0 Days | 3 | 7 | 13 | 15 | 18 | 23 | 26 | 29 | 33 | 36 | 40 | 43 | 48 | 53 | 64 | 68 | 74 | 80 | 89 | 97 | 105 | 110 | 122 | 133 Days |
| --- | --- | --- | --- | --- | --- | --- | --- | --- | --- | --- | --- | --- | --- | --- | --- | --- | --- | --- | --- | --- | --- | --- | --- | --- | --- | --- |
| B1 | 30 | 11.62 | 36.28 | 37.87 | 32.36 | 24.73 | 35.06 | 23.1 | 22.6 | 23.56 | 42.32 | 83.62 | 98.04 | 308 | 294 | 331 | 1147.00 | 2381.00 | 4058.00 | 6629.00 | 6953.00 | 5379.00 | 5479.00 | 2870 | 2265 | 2685 |
| B2 | 30 | 7.69 | 32.73 | 26.54 | 27.65 | 31.27 | 21.27 | 22.01 | 18.86 | 7.78 | 14 | 29.18 | 61.912 | 117 | 52.35 | 281 | 427.00 | 576.00 | 779.00 | 1512.00 | 4076.00 | 5176.00 | 5030.00 | 1955 | 2750 | 2075 |
| B3 | 30 | 7.95 | 75.78 | 45.84 | 76.99 | 57.39 | 49.63 | 34.62 | 29.74 | 56.76 | 113.38 | 124 | 292.28 | 371 | 455 | 737 | 1958.00 | 94.07 | 7249.00 | 4644.00 |  | 6483.00 | 6688.00 | 5750 | 4980 | 5000 |
| B4 | 30 | 13.95 | 26.91 | 21.17 | 26.15 | 22.36 | 18.84 | 15.64 | 15.73 | 12.36 | 10.84 | 12.68 | 15.33 | 45.52 | 123 | 167 | 289.00 | 95.76 | 544.00 | 357.00 | 485.00 | 603.00 | 1400.00 | 2225 | 2435 | 3930 |
| B5 | 30 | 5.01 | 20.61 | 11.55 | 24.69 | 11.37 | 20.65 | 21.12 | 17.55 | 18.65 | 14.23 | 10.95 | 16.43 | 39.2 | 165 | 206 | 346.00 | 97.79 | 141.00 | 264.00 | 425.00 | 491.00 | 573.00 | 160 | 290 | 650 |
| B7 | 30 | 7.37 | 19.79 | 18.18 | 17.19 | 16.72 | 15.53 | 13.46 | 7.42 | 11.58 | 6.23 | 18.82 | 10.56 | 12.95 | 46.87 | 120 | 234.00 | 323.00 | 293.00 | 311.00 | 533.00 | 573.00 | 604.00 | 415 | 530 | 425.25 |
| B8 | 30 | 10.2 | 34.25 | 27.04 | 26.1 | 19.62 | 20.96 | 11.14 | 12.49 | 5.58 | 27.09 | 47.63 | 42.08 | 103 | 222 | 934 | 4324.00 | 572.00 | 462.00 | 692.00 | 1383.00 | 1478.00 | 2582.00 | 870 | 955 | 550 |
| B9 | 20 | 12.86 | 49.16 | 51.01 | 41.1 | 35.08 | 33.24 | 30.39 | 23.07 | 20.42 | 58.79 | 43.11 | 150.32 | 231 | 284 | 549 | 4494.00 | 1305.00 | 1624.00 | 1514.00 | 2790.00 | 2794.00 | 2998.00 | 1100 | 1310 | 2000 |
| B10 | 20 | 9.33 | 39.51 | 19.77 | 29.56 | 26.57 | 17.69 | 19.01 | 17.94 | 17.74 | 12.76 | 12.82 | 38.08 | 51.38 | 52.76 | 131 | 650.00 | 1081.00 | 1620.00 | 3026.00 | 6352.00 | 5220.00 | 5745.00 | 1720 | 1590 | 1060 |
| B11 | 20 | 4.32 | 29.22 | 15.83 | 15.18 | 14.3 | 12.67 | 8.04 | 9.2 | 9.1 | 6.97 | 5.83 | 5.41 | 8.73 | 35.52 | 48.58 | 144.00 | 14.68 | 61.46 | 90.08 | 161.00 | 176.00 | 197.00 | 225 | 310 | 360 |
| B12 | 20 | 7.89 | 15.8 | 9.34 | 18.39 | 17.69 | 15.37 | 14.05 | 6.95 | 7.04 | 8.21 | 6.6 | 7.97 | 33.72 | 63.72 | 136 | 226.00 | 46.02 | 90.98 | 144.00 | 264.00 | 375.00 | 460.00 | 130 | 220 | 570 |
| B13 | 20 | 8.7 | 9.66 | 8.83 | 8.78 | 6.83 | 6.54 | 7.28 | 5.92 | 5.69 | 3.65 | 3.36 | 3.28 | 3.84 | 7.06 | 19.22 | 79.83 | 80.43 | 74.18 | 70.39 | 119.00 | 144.00 | 217.00 | 100 | 145 | 98 |
| B14 | 20 | 3.25 | 11.53 | 11.88 | 11.49 | 10.71 | 9.91 | 6.71 | 7.35 | 7.95 | 5.44 | 6.99 | 4.55 | 39.12 | 23.61 | 42.5 | 194.00 | 40.14 | 51.23 | 112.00 | 243.00 | 240.00 | 281.00 | 60 | 90 | 236.35 |
| B15 | 20 | 4.67 | 20.1 | 18.09 | 17.72 | 15.87 | 15.78 | 13.59 | 12.66 | 12.8 | 8.96 | 13.88 | 19.95 | 6.23 | 72.68 | 123 | 231.00 | 37.47 | 66.03 | 114.00 | 229.00 | 580.00 | 2436.00 | 1635 | 1970 | 620 |
| B16 | 20 | 3.23 | 199.75 | 253 | 1228 | 1022 | 1332 | 1512 | 888 | 1198 | 983 | 803 | 1341 | 1248 | 1097 | 1207 | 1581.00 | 91.95 | 141.00 | 199.00 | 322.00 | 566.00 | 860.00 | 295 | 440 | 420.7 |
| B17 | 20 | 4.1 | 216.8 | 240 | 822 | 617 | 1304 | 1527 | 7.82 | 1035 | 1004 | 881 | 1001 | 698 | 1316 | 1301 | 2963.00 | 10.55 | 287.00 | 218.00 | 376.00 | 423.00 | 623.00 | 120 | 165 | 710 |
| B18 | 20 | 8.9 | 187 | 260 | 1001 | 1399 | 1147 | 1808 | 1256 | 1336 | 887 | 615 | 908 | 1058 | 1568 | 1005 | 2003.00 | 84.66 | 310.00 | 152.00 | 204.00 | 508.00 | 601.00 | 235 | 200 |  |

Experiment 1 – CO2

| Sample | BES | 0 Days | 3 | 7 | 13 | 15 | 18 | 23 | 26 | 29 | 33 | 36 | 40 | 43 | 48 | 53 | 64 | 68 | 74 | 80 | 89 | 97 | 105 | 110 | 122 | 133 Days |
| --- | --- | --- | --- | --- | --- | --- | --- | --- | --- | --- | --- | --- | --- | --- | --- | --- | --- | --- | --- | --- | --- | --- | --- | --- | --- | --- |
| B1 | 30 mM BES | 360 | 1,774 | 2205 | 2551 | 2285 | 3297 | 2606 | 2540 | 3405 | 3800 | 2547 | 1372 | 3058 | 2154 | 2026 | 2078 | 2214 | 2389 | 2453 | 2659 | 2196 | 2387 | 1590 | 1370 | 1800 |
| B2 | 30 mM BES | 272 | 1575 | 1921 | 2357 | 2750 | 2537 | 2548 | 2708 | 1441 | 2252 | 2581 | 2400 | 2353 | 593 | 2196 | 2193 | 2008 | 2085 | 1988 | 1325 | 2319 | 2344 | 2250 | 1615 | 1975 |
| B3 | 30 mM BES | 404 | 4478 | 2002 | 4445 | 5043 | 3899 | 4415 | 4877 | 5202 | 4429 | 3413 | 3695 | 3739 | 3378 | 3675 | 3659 | 1512 | 4669 | 1323 | 4264 | 4116 | 3822 | 2405 | 2425 | 2690 |
| B4 | 30 mM BES | 262 | 2363 | 1996 | 3124 | 2971 | 2690 | 2790 | 3772 | 3823 | 3026 | 3082 | 2425 | 2904 | 2624 | 2406 | 2402 | 2602 | 1790 | 2441 | 2632 | 2497 | 2722 | 2460 | 1585 | 2795 |
| B5 | 30 mM BES | 151 | 1516 | 1100 | 2532 | 1214 | 2323 | 2835 | 2589 | 3407 | 2723 | 2277 | 2405 | 1549 | 2338 | 2141 | 2255 | 2489 | 2119 | 2212 | 2144 | 1954 | 2064 | 775 | 880 | 1870 |
| B7 | 30 mM BES | 254 | 2372 | 2229 | 2668 | 2693 | 3032 | 2936 | 1907 | 3945 | 2073 | 2847 | 2635 | 2535 | 2341 | 2408 | 2115 | 2508 | 2282 | 2049 | 2287 | 1948 | 1890 | 1520 | 1575 | 1575 |
| B8 | 30 mM BES | 217 | 3423 | 2797 | 3362 | 2671 | 3357 | 2142 | 2320 | 1325 | 2444 | 2425 | 2052 | 2519 | 2696 | 2215 | 1702 | 2833 | 2411 | 2282 | 3028 | 1478 | 3095 | 1245 | 1280 | 680 |
| B9 | 20 mM BES | 249 | 3326 | 2957 | 3450 | 2847 | 3690 | 3852 | 3637 | 2558 | 3437 | 2762 | 3025 | 2744 | 2464 | 2623 | 2487 | 2188 | 2269 | 2225 | 2671 | 2533 | 2531 | 1145 | 1210 | 1800 |
| B10 | 20 mM BES | 308 | 3232 | 1410 | 2994 | 2743 | 1791 | 2793 | 2801 | 3417 | 2754 | 2704 | 2704 | 2408 | 2048 | 2061 | 2234 | 2137 | 2068 | 2128 | 2510 | 2188 | 2495 | 2205 | 2430 | 720 |
| B11 | 20 mM BES | 371 | 1874 | 1680 | 1875 | 1945 | 2020 | 1451 | 2447 | 2892 | 2381 | 2260 | 2088 | 2192 | 2061 | 1883 | 1999 | 1897 | 2003 | 1827 | 2061 | 1910 | 1670 | 2170 | 2075 | 2415 |
| B12 | 20 mM BES | 256 | 1681 | 966 | 2339 | 2547 | 2456 | 2585 | 1703 | 2077 | 2775 | 2348 | 2492 | 2245 | 2051 | 2011 | 1961 | 1873 | 1887 | 1815 | 1748 | 1885 | 1933 | 785 | 1030 | 2300 |
| B13 | 20 mM BES | 380 | 1236 | 1175 | 1382 | 1366 | 1364 | 1812 | 1862 | 2135 | 1245 | 1241 | 1284 | 1452 | 1335 | 1324 | 1426 | 1398 | 1275 | 1268 | 1293 | 1151 | 1294 | 695 | 670 | 755 |
| B14 | 20 mM BES | 118 | 1581 | 1658 | 1834 | 1884 | 1971 | 1511 | 2270 | 3081 | 2284 | 2205 | 1432 | 1753 | 1704 | 1595 | 1772 | 1722 | 1592 | 1519 | 1757 | 1509 | 1796 | 415 | 590 | 1460 |
| B15 | 20 mM BES | 390 | 1792 | 1688 | 2109 | 2070 | 2365 | 2323 | 2120 | 2782 | 2212 | 2137 | 1953 | 1993 | 1711 | 1718 | 1909 | 1480 | 1454 | 1650 | 1532 | 1435 | 1630 | 1025 | 1230 | 445 |
| B16 | 0 mM BES | 213 | 1276 | 740 | 587 | 1158 | 1687 | 547 | 1174 | 1552 | 1843 | 1534 | 1849 | 1877 | 1703 | 1868 | 2005 | 1147 | 1631 | 1747 | 1793 | 1849 | 2057 | 820 | 995 | 800 |
| B17 | 0 mM BES | 132 | 1115 | 2335 | 2936 | 2712 | 2114 | 1973 | 3027 | 3348 | 2596 | 2614 | 2515 | 2261 | 1951 | 2127 | 2054 | 1780 | 2001 | 1901 | 1825 | 2040 | 1991 | 1025 | 1005 | 1000 |
| B18 | 0 mM BES | 204 | 1179 | 988 | 1106 | 1353 | 816 | 899 | 1193 | 1985 | 1117 | 1302 | 1807 | 997 | 1242 | 1898 | 1586 | 2130 | 1170 | 1449 | 2130 | 1908 | 2004 | 1055 | 1000 |  |

Experiment 1 – d13CH4

| Sample | BES | 0 Days | 3 | 7 | 13 | 15 | 18 | 23 | 26 | 29 | 33 | 36 | 40 | 43 | 48 | 53 | 64 | 68 | 74 | 80 | 89 | 97 | 105 | 110 | 122 | 133 Days |
| --- | --- | --- | --- | --- | --- | --- | --- | --- | --- | --- | --- | --- | --- | --- | --- | --- | --- | --- | --- | --- | --- | --- | --- | --- | --- | --- |
| B1 | 30 mM BES | -35.8435886 | -44.86472348 | -37.66351552 | -52.48077259 | -43.19855304 | -39.02677844 | -43.73416927 | -87.56915668 | -47.14316595 | -62.97181422 | -60.16127818 | -66.98571698 | -66.6177918 | -72.85807588 | -62.46 | -30.41 | -55.58 | -28.72 | -35.49 | -36.15 | -36.88 | -38.15 | -35.17749138 | -37.41509819 | -35.62 |
| B2 | 30 mM BES | -52.61303642 | -49.10869303 | -48.57310588 | -52.80971002 | -47.41655647 | -51.20048826 | -49.73969433 | -76.39800497 | -50.37918506 | -49.10400076 | -65.73237285 | -86.7540464 | -88.88559191 | -84.56880552 | -79.56498709 | -79.95 | -82.36 | -64.27 | -63.52 | -47.52 | -34.49 | -35.73 | -34.07841498 | -35.57007913 | -35.09 |
| B3 | 30 mM BES | -50.67512808 | -48.36985983 | -49.31692436 | -51.45371664 | -48.55171682 | -47.77866844 | -49.2038059 | -62.2682493 | -53.77607577 | -43.77486429 | -58.70628589 | -78.73459182 | -83.00633185 | -89.69853655 | -80.42725043 | -60.30 | -44.29 | -37.53 | -41.28 |  |  |  | -31.99127 | -34.87696994 | -36.38 |
| B4 | 30 mM BES | -41.8257357 | -52.40120406 | -51.73817381 | -51.34373924 | -50.0145027 | -52.08991942 | -52.08046221 | -57.92854726 | -47.79530421 | -44.72801937 | -46.78043879 | -63.15910588 | -85.71743392 | -93.95066289 | -93.62979747 | -87.74 | -94.32957745 | -47.69 | -61.60 | -67.05 | -66.84 | -55.84 | -44.4899608 | -29.35076951 | -32.64 |
| B5 | 30 mM BES | -48.87089702 | -48.90645382 | -47.45378142 | -48.30573137 | -45.85972366 | -46.32096335 | -47.22207773 | -51.60544841 | -45.27368946 | -41.83251261 | -45.17813122 | -60.2305322 | -69.35269127 | -84.60543792 | -88.51979527 | -91.38 | -96.81 | -99.24 | -95.21 | -98.45 | -96.74 | -97.32 | -91.53362206 | -78.59000771 | -67.09 |
| B7 | 30 mM BES | -52.48261413 | -47.32204727 | -47.41806366 | -43.61845916 | -41.46993874 | -42.50357074 | -41.35072889 | -47.25215359 | -39.87923331 | -39.85303785 | -45.44635354 | -45.49934797 | -60.71275221 | -79.2697359 | -80.99094629 | -82.84 | -86.46 | -86.35 | -89.36 | -92.75 | -91.45 | -94.23 | -91.5351315 |  | -94.32 |
| B8 | 30 mM BES | -46.9713569 | -46.78800426 | -45.10972518 | -44.92206401 | -43.7412246 | -47.99816482 | -45.82835937 | -50.60783335 | -46.91352332 | -50.91706099 | -46.18994797 | -41.27185443 | -68.84375377 | -68.60286879 | -53.03172458 | -22.30 | -25.00 | -34.68 | -40.75 | -43.69 | -46.00 | -42.90 | -40.77663576 |  | -47.84 |
| B9 | 20 mM BES | -50.91542022 | -48.96460551 | -47.06231061 | -46.92442447 | -46.32533853 | -45.5040199 | -45.11764354 | -45.95968005 | -50.24088271 | -46.22745266 | -48.20544139 | -70.52459997 | -72.10770589 | -73.57670965 | -64.64258856 | -31.10 | -20.92 | -32.93 | -38.95 | -41.89 | -43.59 | -45.45 | -42.06438104 |  | -48.36 |
| B10 | 20 mM BES |  |  | -47.30518498 | -47.61634805 | -45.87717731 | -44.60531066 | -45.05609252 | -47.32061691 | -44.51467398 | -41.49342715 | -44.15022149 | -40.02353247 | -49.08957823 | -68.45193326 | -80.30639944 | -57.26 | -57.48 | -54.70 | -47.06 | -26.33 | -27.44 | -28.60 | -25.18913133 |  | -36.71 |
| B11 | 20 mM BES |  |  | -46.00387887 | -44.93111663 | -42.76332456 | -41.85244493 | -42.70474637 | -43.83162215 | -37.7203154 | -37.88146445 | -35.40955067 | -37.32169524 | -57.92659985 | -85.89797638 | -90.97842369 | -96.54 | -94.13 | -89.38 | -97.45 | -97.23 | -98.79 | -93.02 | -96.22852167 |  | -88.65 |
| B12 | 20 mM BES | -52.85274841 | -49.04401998 | -44.32133046 | -45.96258549 | -43.76059793 | -43.2133761 | -41.4191239 | -44.49176302 | -38.47792486 | -34.80700745 | -33.02609549 | -41.89660413 | -77.87292963 | -85.02426359 | -80.1930154 | -89.28 | -96.55 | -100.37 | -99.49 | -95.63 | -99.75 | -98.01 | -95.14935739 |  | -91.06 |
| B13 | 20 mM BES |  |  | -45.35477672 | -41.90805951 | -40.70491947 | -40.90486308 | -40.52362245 | -46.95090046 | -42.1986156 | -42.39060427 | -39.79960915 | -30.68763636 | -46.94174187 | -70.58549565 | -86.37297861 | -98.42 | -99.44 | -97.59 | -95.03 | -101.12 | -100.98 | -100.13 | -94.77764165 |  | -95.95 |
| B14 | 20 mM BES | -52.73565066 | -47.16036517 | -43.52404938 | -42.25873982 | -40.93699279 | -40.98975385 | -39.7599951 | -45.11076153 | -37.0569861 | -35.55602187 | -33.57751591 | -28.81689578 | -83.95474944 | -85.45416976 | -90.74179317 | -81.50 | -99.16 | -101.10 | -93.15 | -100.72 | -100.99 | -100.10 | -93.25163688 |  | -97.60 |
| B15 | 20 mM BES | -52.19164654 | -49.75096548 | -46.79628128 | -45.48369886 | -44.83423686 | -45.04517878 | -42.46880203 | -43.76407155 | -39.15103668 | -36.31739871 | -35.78549661 | -61.77677791 | -40.89291744 | -83.34670339 | -86.18331515 | -89.85 | -94.83 | -96.37 | -101.05 | -82.03 | -60.72 | -39.85 | -25.06036126 |  | -31.32 |
| B16 | 0 mM BES | -52.44524058 | -48.15631928 | -39.88309078 | -35.00998083 | -31.18408036 | -32.41706704 | -34.93405875 | -34.90630638 | -35.42776662 | -35.16399594 | -36.00270425 | -36.07281059 | -36.89087776 | -37.46770959 | -37.97181969 | -39.05 | -48.54 | -47.90 | -46.89 | -46.37 | -43.85 | -43.95 | -38.30652361 |  | -40.47 |
| B17 | 0 mM BES |  |  |  |  |  |  |  |  |  |  |  |  |  |  |  |  |  |  |  |  |  |  |  |  |  |
| B18 | 0 mM BES |  |  |  |  |  |  |  |  |  |  |  |  |  |  |  |  |  |  |  |  |  |  |  |  |  |

Experiment 2 – d13CH4

| Group | Core | Depth | 0 Days | 7 | 14 | 21 | 28 | 35 | 42 | 51 | 59 | 67 | 72 | 84 Days |
| --- | --- | --- | --- | --- | --- | --- | --- | --- | --- | --- | --- | --- | --- | --- |
| 20 mM BES | 2 | 0-8cm | -50.86270107 | -46.23363242 | -19.95617563 | -30.80526136 | -86.30166977 | -89.5673088 | -31.4 | -31.19963328 |  |  |  | -33.4535133 |
| 20 mM BES | 2 | 0-8cm | -40.27106633 | -52.97153013 | -36.65573994 | -15.21492222 | -43.08078973 | -93.6709754 | -33.9 | -57.31784207 | -45.9323023 | -14.31912175 |  | -28.15171396 |
| 20 mM BES | 2 | 0-8cm | -52.30359258 | -50.6509554 | -36.71589503 | -32.54079465 | -90.28492551 | -89.67413362 | -59.7 | -69.15256515 |  |  | -31.09080861 | -34.41796801 |
| 0 mM BES | 2 | 0-8cm | -52.88363256 | -53.89134387 | -48.96107896 | -31.63069224 |  |  | -33.3 |  | -35.74214246 | -38.75413872 | -36.34259393 | -36.95269408 |
| 0 mM BES | 2 | 0-8cm | -51.14397584 | -54.05865433 | -45.9406911 | -28.57231828 | -31.3011635 |  | -33.6 | -35.84754409 | -35.68562008 | -38.12204494 | -36.18363329 | -37.03951466 |
| 20 mM BES | 2 | 8-16cm | -51.59649703 | -48.38153519 | -53.69542289 | -51.03208945 | -72.87836098 | -93.27520115 | -94 | -88.12871288 | -71.12237719 | -41.68384092 | -36.30326329 | -36.90040065 |
| 20 mM BES | 2 | 8-16cm | -53.69510303 | -53.73669129 | -53.16142401 | -36.41849001 | -61.94448918 | -79.56959945 | -91.9 | -83.55865778 | -71.66434493 | -49.21005384 | -35.52499417 | -33.96170501 |
| 20 mM BES | 2 | 8-16cm | -52.89099438 | -50.78890748 | -50.76053644 | -54.98918317 | -88.07502005 | -94.92061842 | -95.8 | -92.25658704 | -85.69615025 | -64.55415886 | -45.50899563 | -34.22660973 |
| 0 mM BES | 2 | 8-16cm | -53.39438694 | -52.94304281 | -52.58658602 | -64.11732974 | -43.53499714 | -38.40592864 | -39.9 | -41.85561987 | -42.10688182 | -43.94302147 | -40.56180413 | -41.78498055 |
| 0 mM BES | 2 | 8-16cm | -51.61090782 | -53.763582 | -62.08467634 | -77.55657069 | -47.87907305 | -41.82866434 | -41.8 | -42.06246572 | -34.00930962 | -44.54034397 | -41.83390789 | -43.45104871 |
| 20 mM BES | 3 | 0-8cm | -52.40324334 | -54.26823798 | -42.89483962 | -38.5056629 | -88.99826759 | -93.25996429 | -93.7 | -70.04506457 | -90.07833349 | -86.88814669 | -81.53365847 | -76.54244068 |
| 20 mM BES | 3 | 0-8cm | -52.82573897 | -51.17896616 | -37.395488 | -37.00104317 | -45.41683452 | -89.02419136 | -61.8 | -90.63468359 | -73.10099935 | -87.89124168 | -83.09087369 | -83.57491757 |
| 20 mM BES | 3 | 0-8cm | -51.83465376 | -52.60803706 | -40.63082962 | -34.72867247 | -87.93244676 | -94.14972418 | -91.8 | -92.25268769 | -91.6382625 | -76.70329882 | -74.08494108 | -47.3255392 |
| 0 mM BES | 3 | 0-8cm | -53.18115246 | -51.83369626 | -43.68821636 | -39.06018841 | -34.19486589 | -36.27773692 | -40.7 | -41.88478591 | -40.03380326 | -42.12103057 | -39.46102206 | -39.46570379 |
| 0 mM BES | 3 | 0-8cm | -54.47517415 | -54.44277701 | -40.87468993 | -25.52788268 | -32.13574769 |  |  |  | -16.20961254 | -39.08117676 | -36.5137238 | -37.40429462 |
| 20 mM BES | 3 | 8-16cm | -52.89372098 | -51.8123182 | -50.70421299 | -52.71500398 | -74.61518304 | -86.9974753 | -86 | -81.4 | -72.3 | -55.9 | -43.15822383 | -36.9 |
| 20 mM BES | 3 | 8-16cm | -52.43331562 | -52.34916639 | -52.35028847 | -51.83696231 | -63.44317316 | -79.60235151 | -90.1 | -88.6 | -84 | -78 | -69.43929691 |  |
| 20 mM BES | 3 | 8-16cm | -52.62801759 | -51.52391138 | -52.33806773 | -40.64008995 | -77.55891532 | -85.98691917 | -87.3 | -78.6 | -68.6 | -54.6 | -40.35393668 | -37.1 |
| 0 mM BES | 3 | 8-16cm | -51.93381654 | -52.21772604 | -50.17529581 | -48.35086794 | -49.7086098 | -42.70680823 | -40.4 | -42.9 | -44.7 | -45.3 | -42.43031859 | -46.8 |
| 0 mM BES | 3 | 8-16cm | -50.99519098 | -52.18555479 | -48.06926929 | -46.24907206 | -39.71092571 | -40.40442967 | -41.2 |  | -43.2 | -44.5 |  | -45.6 |

Experiment 2 - Methane

| Group | Core | Depth | 0 Days | 7 | 14 | 21 | 28 | 35 | 42 | 51 | 59 | 67 | 72 | 84 Days |
| --- | --- | --- | --- | --- | --- | --- | --- | --- | --- | --- | --- | --- | --- | --- |
| 20 mM BES | 2 | 0-8cm | 4.39 | 5 | 8.18 | 14.44 | 374 | 854 | 2158 | 8672 | 6011 | 6829 | 11885 | 21455 |
| 20 mM BES | 2 | 0-8cm | 9.36 | 4.83 | 19.01 | 5.52 | 10.62 | 251 | 1542 | 1477 | 1394 | 4380 | 6940 | 18345 |
| 20 mM BES | 2 | 0-8cm | 4.39 | 7.1 | 10.34 | 9.11 | 642 | 1090 | 1749 | 2662 | 6547 | 6172 | 7375 | 34770 |
| 0 mM BES | 2 | 0-8cm | 5.75 | 8.61 | 989 | 6749 | 7523 | 4141 | 9508 | 9490 | 8891 | 8253 | 4545 | 5000 |
| 0 mM BES | 2 | 0-8cm | 3.88 | 4.99 | 810 | 5134 | 5520 | 5866 | 6701 | 6760 | 5605 | 4404 | 4115 | 3565 |
| 20 mM BES | 2 | 8-16cm | 7.28 | 6.32 | 12.98 | 14.19 | 26.17 | 168 | 262 | 440 | 805 | 3087 | 1910 | 2250 |
| 20 mM BES | 2 | 8-16cm | 13.88 | 16.34 | 19.51 | 34.87 | 33.88 | 170 | 236 | 501 | 885 | 2825 | 3465 | 2730 |
| 20 mM BES | 2 | 8-16cm | 12.13 | 10.27 | 17.85 | 11.34 | 92.6 | 288 | 307 | 547 | 701 | 1549 | 1970 | 3155 |
| 0 mM BES | 2 | 8-16cm | 10.45 | 13.01 | 49.6 | 148 | 2624 | 3491 | 2962 | 3246 | 3772 | 3685 | 1660 | 2030 |
| 0 mM BES | 2 | 8-16cm | 4.86 | 14.61 | 54.74 | 222 | 2898 | 3829 | 3478 | 3885 | 2990 | 5083 | 2505 | 3005 |
| 20 mM BES | 3 | 0-8cm | 12.41 | 15.72 | 15.52 | 15.47 | 481 | 897 | 951 | 998 | 1215 | 2661 | 1245 | 1350 |
| 20 mM BES | 3 | 0-8cm | 9.72 | 4.58 | 8.55 | 8.56 | 15.68 | 216 | 917 | 1184 | 1022 | 5740 | 6090 | 4285 |
| 20 mM BES | 3 | 0-8cm | 10.38 | 13 | 12.9 | 14.5 | 251 | 587 | 657 | 845 | 941 | 1127 | 415 | 640 |
| 0 mM BES | 3 | 0-8cm | 16.72 | 10.86 | 279 | 1360 | 1792 | 1683 | 1995 | 2572 | 2658 | 1553 | 615 | 700 |
| 0 mM BES | 3 | 0-8cm | 21.17 | 21.85 | 1481 | 5891 | 4698 | 1552 | 7606 | 6579 | 6912 | 1142 | 440 | 1095 |
| 20 mM BES | 3 | 8-16cm | 51.79 | 41.46 | 40.28 | 33.62 | 100 | 237 | 414 | 770 | 1153 | 2718 | 1490 | 1410 |
| 20 mM BES | 3 | 8-16cm | 42.66 | 55.62 | 54.75 | 43.45 | 66.28 | 135 | 308 | 588 | 865 | 1336 | 660 |  |
| 20 mM BES | 3 | 8-16cm | 31.99 | 30.01 | 38.65 | 70.31 | 114 | 386 | 595 | 954 | 1694 | 2735 | 1650 | 980 |
| 0 mM BES | 3 | 8-16cm | 30.47 | 34.14 | 104 | 169 | 1843 | 2827 | 1307 | 2910 | 3113 | 4154 | 1185 | 1765 |
| 0 mM BES | 3 | 8-16cm | 38.25 | 48.02 | 201 | 358 | 4450 | 4253 | 4806 | 2561 | 6157 | 6719 | 2140 | 3775 |

Experiment 2 – CO2

| Group | Core | Depth | 0 Days | 7 | 14 | 21 | 28 | 35 | 42 | 51 | 59 | 67 | 72 | 84 Days |
| --- | --- | --- | --- | --- | --- | --- | --- | --- | --- | --- | --- | --- | --- | --- |
| 20 mM BES | 2 | 0-8cm | 760 | 925 | 4839 | 4750 | 5606 | 5825 | 5992 | 6314 | 7148 | 3561 | 5560 |  |
| 20 mM BES | 2 | 0-8cm | 635 | 978 | 3880 | 506 | 5011 | 4920 | 5636 | 5284 | 4250 | 4916 | 3325 |  |
| 20 mM BES | 2 | 0-8cm | 583 | 1172 | 5095 | 5721 | 6310 | 5537 | 4898 | 6200 | 5959 | 6708 | 3220 |  |
| 0 mM BES | 2 | 0-8cm | 862 | 1150 | 4484 | 5134 | 5985 | 6691 | 6118 | 4068 | 7066 | 5522 | 3355 | 3340 |
| 0 mM BES | 2 | 0-8cm | 553 | 863 | 3805 | 2552 | 4382 | 4511 | 5299 | 5153 | 4420 | 4584 | 3355 | 2815 |
| 20 mM BES | 2 | 8-16cm | 577 | 642 | 2363 | 2681 | 3028 | 2934 | 2721 | 2648 | 2695 | 2750 | 1575 | 1995 |
| 20 mM BES | 2 | 8-16cm | 745 | 965 | 2737 | 2541 | 3413 | 3070 | 2764 | 2788 | 2916 | 2983 | 2375 | 1600 |
| 20 mM BES | 2 | 8-16cm | 716 | 841 | 2349 | 2505 | 3361 | 3195 | 2428 | 1145 | 2873 | 2997 | 1860 | 2340 |
| 0 mM BES | 2 | 8-16cm | 575 | 842 | 2210 | 1769 | 3227 | 3061 | 2981 | 3014 | 3272 | 2969 | 2220 | 2280 |
| 0 mM BES | 2 | 8-16cm | 487 | 898 | 2265 | 1769 | 3022 | 3091 | 3068 | 2520 | 5936 | 3178 | 2060 | 2115 |
| 20 mM BES | 3 | 0-8cm | 737 | 1133 | 4273 | 4968 | 5530 | 5399 | 1116 | 4100 | 4217 | 4419 | 1725 | 2180 |
| 20 mM BES | 3 | 0-8cm | 847 | 1190 | 4647 | 5470 | 5648 | 5493 | 5184 | 4458 | 4556 | 5279 | 2145 | 2070 |
| 20 mM BES | 3 | 0-8cm | 677 | 674 | 3690 | 3968 | 4677 | 4410 | 4717 | 4905 | 3471 | 4140 | 1770 | 1155 |
| 0 mM BES | 3 | 0-8cm | 458 | 578 | 2373 | 3099 | 3528 | 3468 | 3844 | 4009 | 3659 | 3645 | 1780 | 1665 |
| 0 mM BES | 3 | 0-8cm | 912 | 1150 | 4837 | 4943 | 7331 | 7003 | 6595 | 6031 | 6459 | 5470 | 2440 | 2435 |
| 20 mM BES | 3 | 8-16cm | 838 | 864 | 2257 | 2715 | 4159 | 3175 | 3164 | 2917 | 2695 | 3199 | 1640 | 1155 |
| 20 mM BES | 3 | 8-16cm | 756 | 904 | 2217 | 2733 | 3467 | 3436 | 3238 | 3225 | 1970 | 2084 | 1585 | 165 |
| 20 mM BES | 3 | 8-16cm | 709 | 648 | 2203 | 2861 | 3336 | 3403 | 3485 | 2961 | 3232 | 2110 | 1325 | 660 |
| 0 mM BES | 3 | 8-16cm | 587 | 743 | 2042 | 2810 | 3671 | 3417 | 1731 | 3373 | 3228 | 3878 | 2325 | 1575 |
| 0 mM BES | 3 | 8-16cm | 557 | 831 | 2123 | 3008 | 1878 | 2185 | 3512 | 1340 | 3700 | 4119 | 1830 | 2430 |

## Supplementary Figures

**Supplementary Figure 1.** For DNA concentrations, * denotes concentration above the instrument’s detection range and X means it was below the detection limit. Porosity error bars denote two replicates measured once each. All extractions were done with close to 1 g of sediment, so this is also approximately the ng of DNA per g of wet sediment.


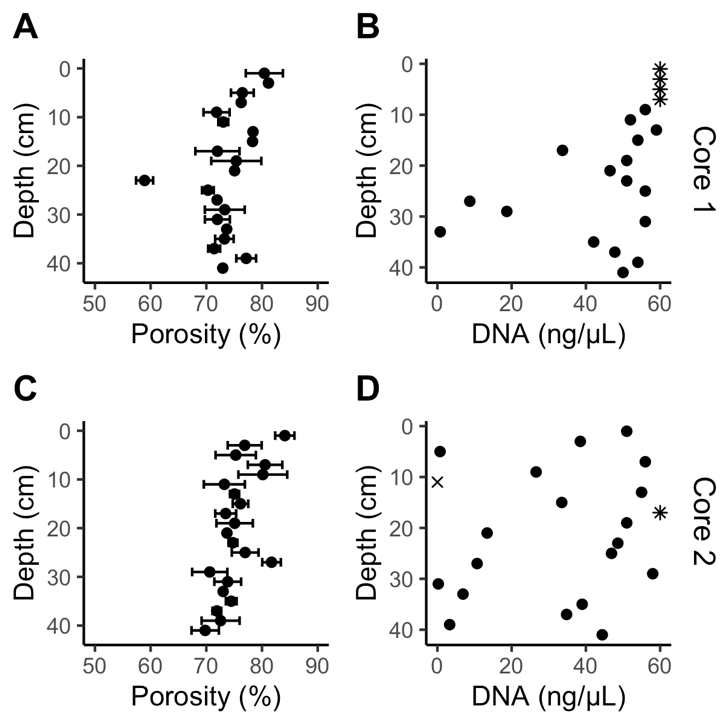


**Supplementary Figure 2.** Alpha diversity values (Chao1, Shannon, Simpson) of Cape Lookout Bight cores, with average Shannon index values of 8.06.


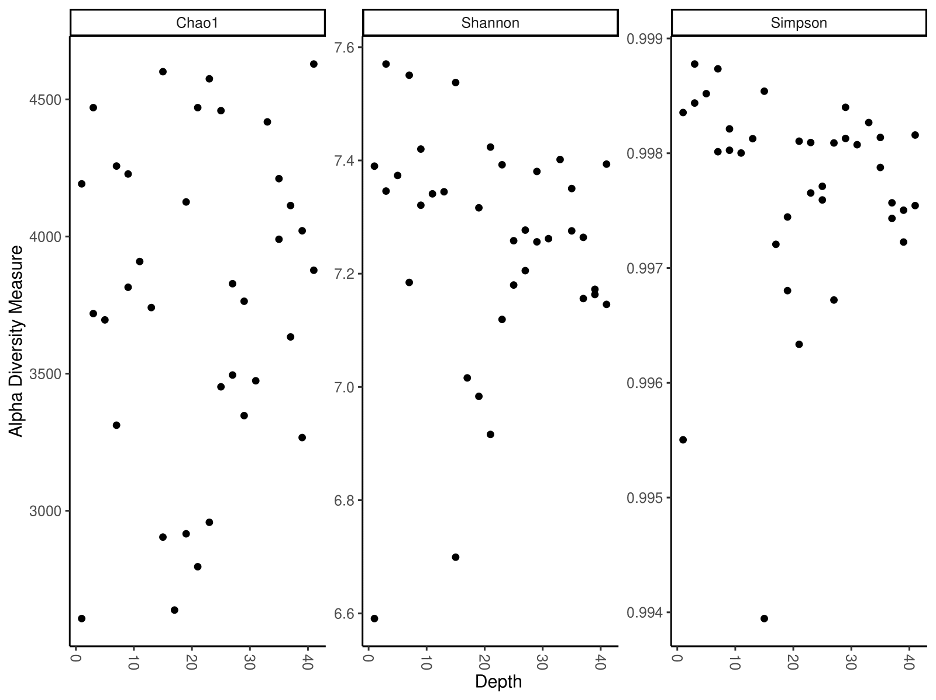


**Supplementary Figure 3.** Summary of core 1 (top plot) and core 2 (bottom plot) for Phyla with > 1% abundance.


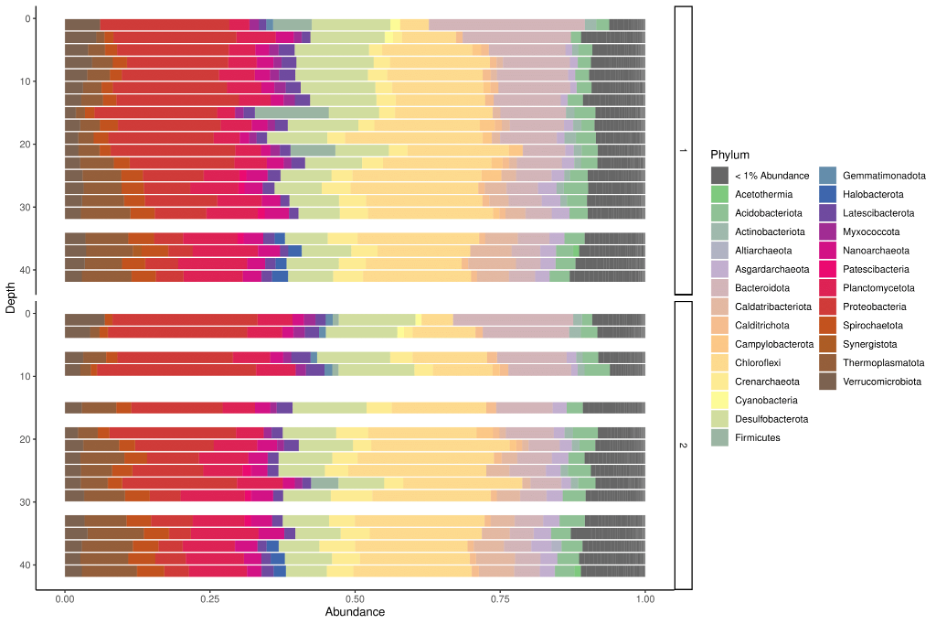


**Supplementary Figure 4.** Heatmap of ANME, methanogens, and SRB for the SRZ (left side) and SMTZ (right side), where ANME-3 is seen correlating with hydrogenotrophic methanogens, and no significant correlation is found between any SRB and ANME archaea.


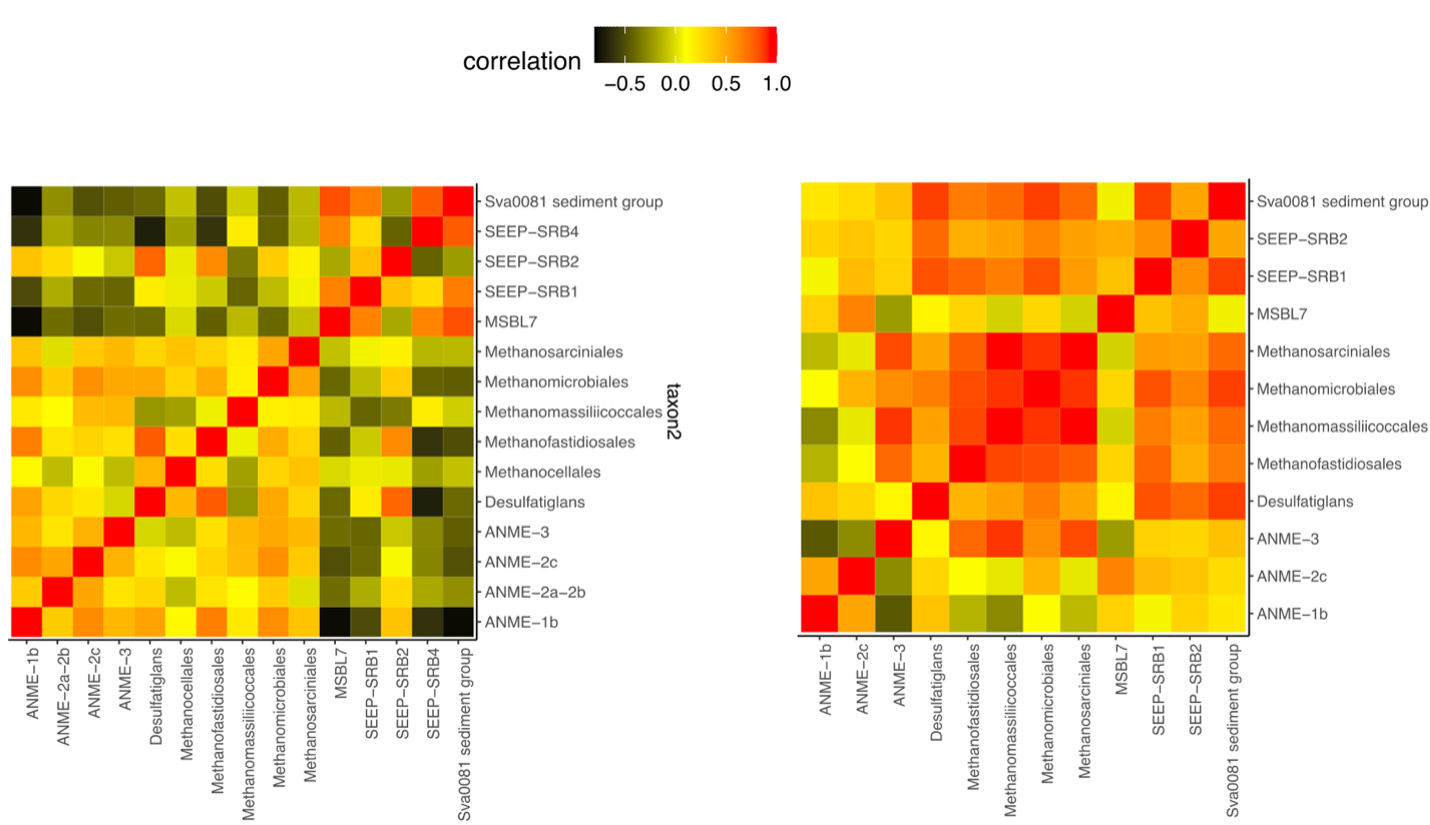


**Supplementary Figure 5.** Linear model of the relationship between downcore sulfide and sulfate in mM. Residuals for sulfide versus sulfate had a minimum of -1.14742, first quartile of -0.21889, median of 0.01625, third quartile of 0.19720, and a max of 0.87451. Residual standard error is 0.385 on 36 degrees of freedom. Multiple R^2^ is 0.7487 with an adjusted R^2^ of 0.7418. T-value is -10.36. Relationship is plotted with a y-intercept of 3.68265 and a slope of -0.13953. P-value is 2.412e-12.
